# A novel protein SPECC1-415aa encoded by N6-methyladenosine modified circSPECC1 regulates the sensitivity of glioblastoma to TMZ

**DOI:** 10.1186/s11658-024-00644-z

**Published:** 2024-09-27

**Authors:** Cheng Wei, Dazhao Peng, Boyuan Jing, Bo Wang, Zesheng Li, Runze Yu, Shu Zhang, Jinquan Cai, Zhenyu Zhang, Jianning Zhang, Lei Han

**Affiliations:** 1 Tianjin Neurological Institute, Key Laboratory of Post-Neuroinjury Neuro-Repair and Regeneration in Central Nervous System, Ministry of Education and Tianjin City, Tianjin Medical University General Hospital, 154 Anshan Road, Heping District, Tianjin, 300052 China; 2 Department of Neurosurgery, The First Affiliated Hospital of Zhengzhou University, Jian She Dong Road 1, Zhengzhou, 480082 Henan Province China; 3 Department of Neurosurgery, The Second Affiliated Hospital of Harbin Medical University, 246 Xuefu Road, Nangang District, Harbin, 150086 China

**Keywords:** Glioblastoma, circSPECC1, Protein encoded, Chemoresistance, m^6^A modification

## Abstract

**Background:**

Circular RNAs (circRNAs) can influence a variety of biological functions and act as a significant role in the progression and recurrence of glioblastoma (GBM). However, few coding circRNAs have been discovered in cancer, and their role in GBM is still unknown. The aim of this study was to identify coding circRNAs and explore their potential roles in the progression and recurrence of GBM.

**Methods:**

CircSPECC1 was screened via circRNAs microarray of primary and recurrent GBM samples. To ascertain the characteristics and coding ability of circSPECC1, we conducted a number of experiments. Afterward, through in vivo and in vitro experiments, we investigated the biological functions of circSPECC1 and its encoded novel protein (SPECC1-415aa) in GBM, as well as their effects on TMZ sensitivity.

**Results:**

By analyzing primary and recurrent GBM samples via circRNAs microarray, circSPECC1 was found to be a downregulated circRNA with coding potential in recurrent GBM compared with primary GBM. CircSPECC1 suppressed the proliferation, migration, invasion, and colony formation abilities of GBM cells by encoding a new protein known as SPECC1-415aa. CircSPECC1 restored TMZ sensitivity in TMZ-resistant GBM cells by encoding the new protein SPECC1-415aa. The m^6^A reader protein IGF2BP1 can bind to circSPECC1 to promote its expression and stability. Mechanistically, SPECC1-415aa can bind to ANXA2 and competitively inhibit the binding of ANXA2 to EGFR, thus resulting in the inhibition of the phosphorylation of EGFR (Tyr845) and its downstream pathway protein AKT (Ser473). In vivo experiments showed that the overexpression of circSPECC1 could combine with TMZ to treat TMZ-resistant GBM, thereby restoring the sensitivity of TMZ-resistant GBM to TMZ.

**Conclusions:**

CircSPECC1 was downregulated in recurrent GBM compared with primary GBM. The m6A reader protein IGF2BP1 could promote the expression and stability of circSPECC1. The sequence of SPECC1-415aa, which is encoded by circSPECC1, can inhibit the binding of ANXA2 to EGFR by competitively binding to ANXA2 and inhibiting the phosphorylation of EGFR and AKT, thereby restoring the sensitivity of TMZ-resistant GBM cells to TMZ.

**Supplementary Information:**

The online version contains supplementary material available at 10.1186/s11658-024-00644-z.

## Background

In the central nervous system, glioblastoma (GBM) is the most prevalent primary malignant tumor [1, 2]. Currently, surgical resection, postoperative chemotherapy and radiation of the tumor within the maximum safe range are frequently used in clinical treatment; however, these methods still fail to achieve ideal results, and approximately 90% of patients relapse [3–5]. Elucidating the molecular mechanism of glioma is highly important for understanding its occurrence, development and recurrence. Recurrence and chemotherapy resistance are major obstacles to achieving satisfactory clinical outcomes in patients with GBM; however, the underlying mechanisms of this phenomenon remain unclear.

Circular RNAs (circRNAs) have been the subject of extensive discussion in the field of cancer in recent years [6, 7]. Numerous studies have demonstrated that abnormally expressed circRNAs play a vital role in glioma progression and can regulate the pathological process of tumors through different mechanisms [8]. CircRNAs, which represent a subclass of noncoding RNAs (ncRNAs), generate covalent single-stranded loops via back-splicing [9]. Several investigations have demonstrated the significant role of circRNAs in a variety of human tumors, including GBM. CircRNAs can act as scaffolds for RNA-binding proteins or as microRNA (miRNA) sponges in multiple tumors [10, 11]. Several circRNAs, including circLRFN5, circNDC80, circASAP1, circMMD and circRNF10, have been shown to play crucial roles in the growth, tumorigenesis, migration and metabolism of GBM [10, 12–15].

CircRNAs have long been regarded as noncoding RNAs owing to their lack of the conventional components that are necessary for cap-dependent translation, such as the poly (A) tail and 5′ cap [16]. Nonetheless, increasing research indicates that certain circRNAs may encode peptides or proteins via cap-independent translation pathways, such as internal ribosomal entry site (IRES) and N6-methyladenosine (m^6^A) modifications [17]. Research has demonstrated that circRNAs can influence the malignant characteristics of tumors, such as proliferation, migration, invasion, and chemoradiotherapy resistance, through encoded proteins or peptides [18]. For example, Wu et al. reported that circSMO encodes a new protein known as SMO-193aa, which is essential for the Hedgehog signaling pathway and can drive glioblastoma genesis, thus suggesting that SMO-193aa is a new target for glioblastoma treatment [19]. Song et al. reported that circHEATR5B encodes a new protein known as HEAT5bB-881aa, which directly interacts with JMJD5 and destabilizes it by phosphorylating S361, increasing pyruvate kinase M2 (PKM2) enzymatic activity in GBM cells, and inhibiting glycolysis and proliferation to inhibit GBM growth [20]. In addition, Duan et al. demonstrated that circMAP3K4 modified by m^6^A can encode a new protein known as MAP3K4-455 aa. By interacting with AIF, MAP3K4-455aa shielded it from cleavage and decreased its amount in the nucleus, thus resulting in cisplatin resistance by preventing cisplatin-induced apoptosis in liver cancer cells [21]. Nonetheless, there are limited studies on the relationship between the coding ability of circRNAs and chemosensitivity of GBM to TMZ. Thus, it is crucial to continue researching the circRNA mechanism and its encoded products in GBM chemosensitivity to TMZ.

Our research demonstrated that circSPECC1 is downregulated in recurrent GBM and encodes a protein of 415 amino acids (aa), which is known as SPECC1-415aa. CircSPECC1 can restrain GBM cell proliferation, migration, invasion, and colony formation abilities by encoding the new protein SPECC1-415aa. The m^6^A reader protein IGF2BP1 can promote the expression and stability of circSPECC1. The sequence of SPECC1-415aa, which is encoded by circSPECC1, can inhibit the binding of ANXA2 to EGFR by competitively binding to ANXA2 and inhibiting the phosphorylation of EGFR and AKT. Moreover, SPECC1-415aa can reverse the sensitivity of TMZ-resistant GBM cells to TMZ. CircSPECC1 can be used as a potential molecular target to suppress the growth of recurrent GBM and reverse the resistance of TMZ-resistant GBM cells to TMZ, thus preventing GBM recurrence.

## Materials and methods

Detailed protocols are provided in the Supplementary materials and the full raw results of the western blot experiments are shown in original images of western blot.

### Acquisition of glioblastoma (GBM) samples

Four primary human glioblastoma (GBM) tissue specimens and four recurrent human GBM tissue specimens that are used for circRNAs microarray were obtained from patients who underwent surgery at the First Affiliated Hospital of Zhengzhou University. Nine recurrent GBM samples and 32 primary GBM samples for circSPECC1 expression level and correlation analysis between circSPECC1 and IGF2BP1 were also obtained from the First Affiliated Hospital of Zhengzhou University. The World Health Organization (WHO) criteria were used by neuropathologists to grade all of the tissue samples. This study was performed in accordance with the principles of the Declaration of Helsinki. The Human Scientific.

Ethics committee of the First Affiliated Hospital of Zhengzhou University approved the study (approval number: 2019-KY-176), and all of the patients provided written informed consent. The clinical data of the GBM samples are available in Supplementary Table 1.

### Cell culture

The 293 T (CRL-3216), U251 (HTX1725), A172 (CRL-1620), SNB19 (HTX2394), U87 (HTB-14), T98G (CRL-1690), and LN229 (CRL-2611) cell lines used in this study were obtained from the American Type Culture Collection (ATCC, Manassas, VA, USA) and long-term cultured at the Institute of Neurology, Tianjin Medical University General Hospital. Cell lines were subjected to STR cell identification and mycoplasma detection. The human immortalized astrocyte cell line UC2 was maintained by our lab and was used as the nontumoral control. UC2 was cultured in AM medium (Astrocyte Medium, ScienCell). Dulbecco’s modified Eagle medium (DMEM, Gibco, USA) was used to cultivate human embryonic kidney cell line 293 T and A172, U87, U251, SNB19, T98G, and LN229 GBM cell lines supplemented with 10% fetal bovine serum (FBS, BI Serum, Israel) in a 37 °C constant temperature incubator (5% CO_2_).

### Construction of TMZ-resistant cell lines

The GBM cell lines (LN229 and U251) and corresponding TMZ-resistant GBM cell lines (LN229R and U251R) that were used for temozolomide (TMZ) sensitivity-related experiments were provided by the research group of Professor Jinquan Cai (Harbin Medical University). The conditions for inducing TMZ resistance were as follows: 6 × 10^3^ LN229 and U251 cells were seeded into 96-well plates. The cell survival rate was determined by using the CCK-8 assay to determine the half maximal inhibitory concentration (IC50) of TMZ. LN229 and U251 (2 × 10^5^) cells were seeded in six-well plates, and the cell culture medium was added at an IC50 of 1/50. When cell growth stabilized, the dose of TMZ was increased. Cultures were preserved for 15 days after each dose increase until the 5th month to the end of induction. The induced TMZ-resistant GBM cell lines were named LN229R and U251R [22].

### Statistical analysis

Photoshop CS6 was the image processing program used in this investigation, and Graphpad Prism 8 was utilized for statistical computation and image creation. Differences between two groups were compared using an unpaired *t* test; additionally, one-way ANOVA was used to compare groups within groups, and two-way ANOVA was used to compare groups within the CCK-8 assay. A log-rank test was used to analyze survival. Differences were considered to be statistically significant if *P* < 0.05.

## Results

### Screening of translated and abnormally expressed circRNAs in GBM

**Fig. 1:**
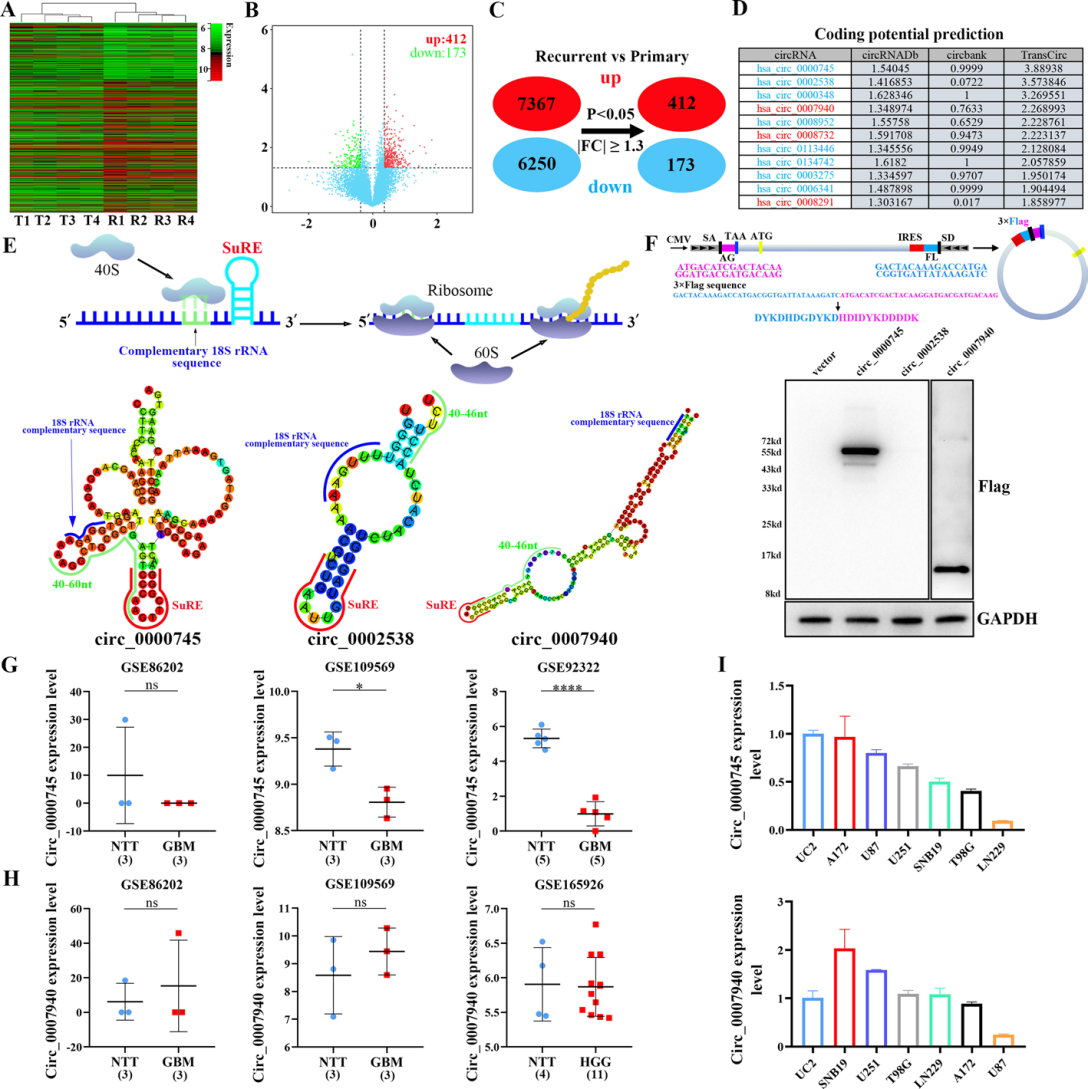
Screening of translated and abnormally expressed circRNAs in GBM. **A** Heatmap of the expression of circRNAs in recurrent and primary GBM samples. **B** Volcano plot of dysregulated circRNAs in recurrent and primary GBM samples. **C** Screening of primary and recurrent GBM samples for differentially expressed circRNAs (|FC|≥ 1.3 and *P* < 0.05). **D** CircRNADb, circbank and TransCirc databases were applied to forecast the coding potential of the top ten upregulated and top ten downregulated circRNAs. **E** Upper: schematic representation of the interaction of 18S rRNA on the ribosomal 40S subunit with the complementary sequence of 18S on the IRES. Lower: secondary structure analysis of three candidate circRNAs with coding potential and their complementary sequences of 18S rRNA and SuRE structural elements. **F** Upper: schematic representation of p-circRNA-Flag plasmids construction for verifying of coding abilities of three candidate circRNAs. Lower: the coding abilities were verified by western blot experiment after transfection of p-circRNA-Flag plasmids in 293 T cells. **G** The circ_0000745 expression levels in non-tumor brain tissues (NTT) and glioblastoma (GBM) were analyzed via GSE86202, GSE109569 and GSE92322 datasets. **H** The circ_0007940 expression levels in NTT and GBM or high-grade glioma (HGG) were analyzed via GSE86202, GSE109569 and GSE165926 datasets. **I** Analysis of circ_0000745 and circ_0007940 expression in GBM cell lines (A172, U87, U251, B19, T98G, and LN229) and normal astrocytes (UC2)

To explore differentially expressed circRNAs with coding potential in tissues from patients with primary GBM and recurrent GBM, we performed circRNAs microarray analysis of 4 primary GBM samples and 4 recurrent GBM samples after standardized therapy. Through circRNAs microarrayanalysis, a total of 7367 circRNAs were found to be upregulated in recurrent GBM compared with primary GBM, and 6250 circRNAs were downregulated in recurrent GBM compared with primary GBM. According to the filter conditions |FC|≥ 1.3 and *P* < 0.05, 412 significantly upregulated circRNAs and 173 significantly downregulated circRNAs were identified via circRNAs microarrayin recurrent GBM compared with primary GBM (Fig. 1A, B). Figure 1C shows a schematic of the screening of differentially expressed circRNAs. Furthermore, the top ten downregulated circRNAs and the top ten upregulated circRNAs were selected according to their FC values, and a total of 20 circRNAs were used for subsequent analysis. The CircRNADb, circBank and TransCirc databases were used to explore the coding potential of the top 20 circRNAs identified above. Owing to the fact that the differences between the circRNADb and circBank scores were small, the ranking was mainly based on the TransCirc coding potential score. The results demonstrated the top 11 circRNAs with coding potential, including hsa_circ_0000745, hsa_circ_0002538, hsa_circ_0000348, hsa_circ_0007940, hsa_circ_0008950, hsa_circ_0008732, hsa_circ_0113446, hsa_circ_0134742, hsa_circ_0003275, hsa_circ_0006341 and hsa_circ_0008291 (Fig. 1D). Two downregulated circRNAs (hsa_circ_0000745 and hsa_circ_0002538), and one upregulated circRNA (hsa_circ_0007940) with the highest coding potential scores were selected for further coding potential analysis.

Recent studies have demonstrated that the complementary sequence of 18S rRNA and the 40–60 nt SuRE on circRNA IRESs are important for promoting the translational initiation of endogenous circRNAs [23]. The SuRE may cause a pause in RNA unwinding, thereby increasing the chance that complementary 18S rRNA sequences on the IRES interact with 18S rRNA on the ribosome and consequently facilitating circRNA translation in a cap-independent manner (Fig. 1E upper). Therefore, the characteristics of the structural elements (18S rRNA complementary sequence and SuRE) in the IRES sequences of the abovementioned three circRNAs were analyzed via RNAfold WebServer. The results showed that there were 18S rRNA complementary sequences and SuRE elements in the circ_0000745, circ_0002538 and circ_0007940 IRESs, and all of the 18S rRNA complementary sequences occurred before the SuRE element. These results indicated that the IRESs of circ_0000745, circ_0002538 and circ_0007940 may encode proteins (Fig. 1E, lower). To further explore whether circ_0000745, circ_0002538, and circ_0007940 encode proteins or peptides, p-circRNA-Flag plasmids with 3 × Flag tag sequences were constructed (Fig. 1F, upper panel). The splicing site was located between the 3 × Flag tag sequences. Only when translation across the splice site was met, the plasmid could translate and express the complete Flag protein. The results showed that there were no Flag protein bands in the vector and p-circ_0002538-Flag groups, whereas p-circ_0000745-Flag and p-circ_0007940-Flag had a Flag protein band, and the protein size was consistent with the predicted size of the online website, thus suggesting that circ_0000745 and circ_0007940 both had the ability to encode proteins (Fig. 1F lower).

Compared with that in primary GBM samples, the circ_0000745 expression level was downregulated, and the circ_0007940 expression level was upregulated, in recurrent GBM samples. Afterwards, the expression levels of circ_0000745 and circ_0007940 in nontumor brain tissues (NTTs) and GBM or high-grade glioma (HGG) tissues were analyzed through four GEO datasets, including GSE86202, GSE109569, GSE92322, and GSE165926. The results showed that circ_0000745 expression was downregulated in patients with GBM compared with in patients with NTT in the GSE109569, GSE92322, and GSE92322 datasets (Fig. 1G). In contrast, circ_0007940 expression did not significantly differ between NTT and GBM or HGG in the GSE86202, GSE109569, and GSE165926 datasets (Fig. 1H). The results of the expression level analysis of various cell lines also demonstrated that circ_0000745 was highly expressed in UC2 cells and that it was expressed at considerably lower levels in GBM cell lines (A172, U87, U251, SNB19, T98G, and LN229), which is consistent with the abovementioned three GEO database analyses. However, the expression level of circ_0007940 had no obvious trend in UC2 or GBM cell lines, which was consistent with the results of the GEO database analysis (Fig. 1I). Therefore, circ_0000745, which has both coding potential and abnormal expression, was selected as the research object.

### CircSPECC1 characteristics in GBM

**Fig. 2:**
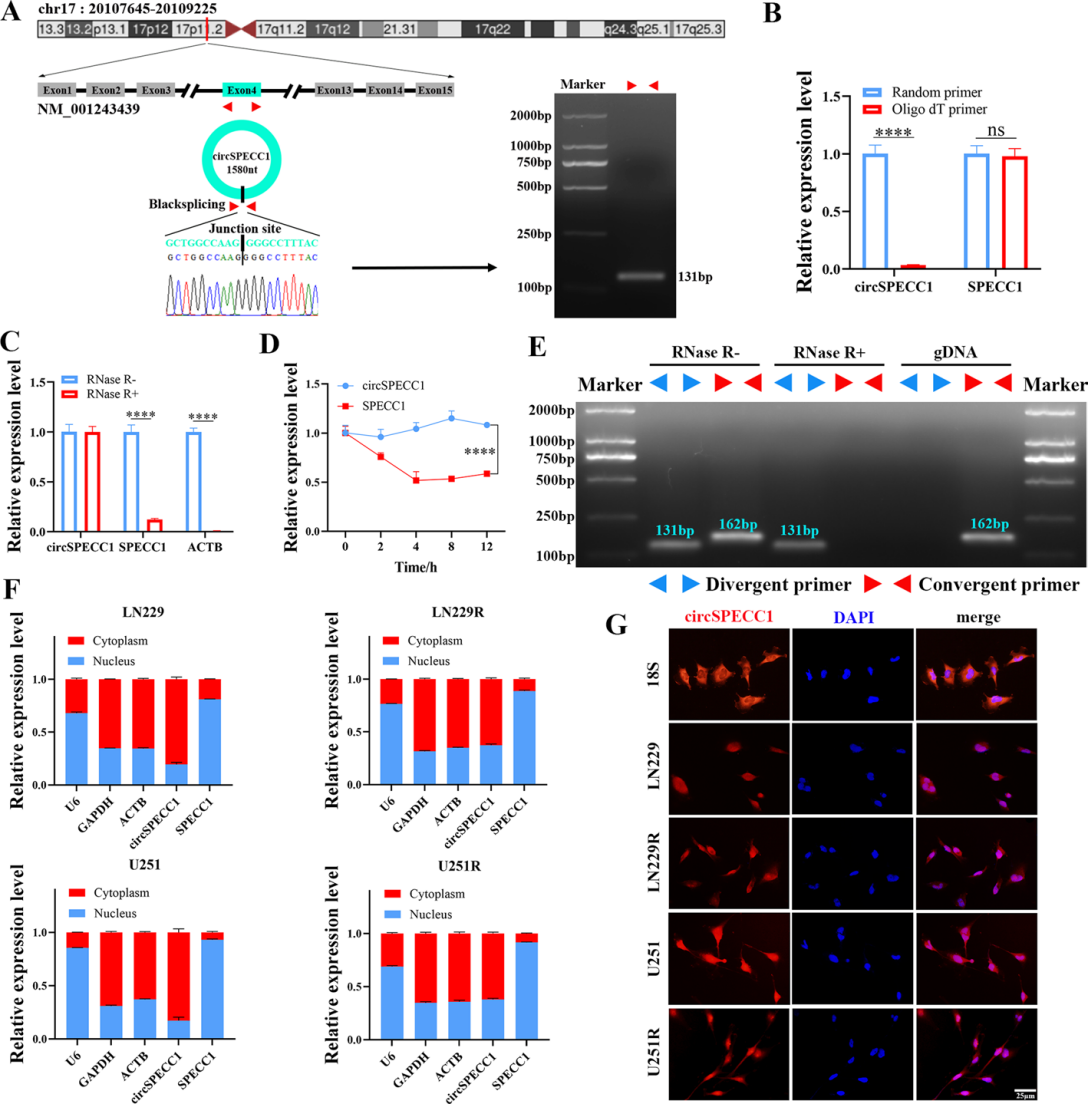
Validation of the circular structure and subcellular localization of circSPECC1. **A** The location of circSPECC1 in the human genome (upper panel). Schematic diagram of circSPECC1 structure and Sanger sequencing to verify the splice site of circSPECC1 (left panel). The specificity of the divergent primers and product size were checked by agarose gel electrophoresis following quantitative reverse transcription polymerase chain reaction (RT-qPCR) (right panel). **B** RT-qPCR analysis of circSPECC1 expression after amplification with random primer or oligo (dT) primer. **C** RT-qPCR analysis of circSPECC1 expression after treated with RNase R. **D** CircSPECC1 expression detected by RT-qPCR after Act D treatment. **E** The presence of circSPECC1 in cDNA and gDNA samples from 293T cells was detected using divergent and convergent primer. **F** The distribution of circSPECC1 examined by cytoplasmic and nuclear RNA isolation. GAPDH and U6 in the cytoplasm and nucleus were used as positive controls, respectively. **G** CircSPECC1 distribution in the cytoplasm and nucleus was demonstrated by RNA fluorescence in situ hybridization. Every experiment was carried out a minimum of three times, and the mean ± standard deviation (SD) of the results was given. ns, *P* > 0.05; ****, *P* < 0.0001

According to the circBase database, hsa_circ_0000745 is located on the short arm of chromosome 17. It is formed via the splicing of exon 4 of the 15 exons of the NM_001243439 transcript that is transcribed by the parental gene sperm antigen with calponin homology and coiled-coil domains 1 (*SPECC1*). With a size of 1580 nt, it was named circSPECC1 (Fig. 2A). We used Sanger sequencing to confirm the specific back-splicing junction site sequence to confirm that circSPECC1 was circular (Fig. 2A, left panel). Agarose gel electrophoresis was used to determine the specificity and product size of the divergent circSPECC1 primer that was used to amplify the back-splicing junction site (Fig. 2A, right panel). The results showed that circSPECC1 could be amplified by this divergent primer with great specificity, and the product size was as expected. SPECC1 mRNA was amplified by both random primers and oligo (dT) primers; however, circSPECC1 could not be amplified by oligo (dT) primers compared with random primers (Fig. 2B). Furthermore, circSPECC1 demonstrated greater resistance to RNase R than SPECC1 mRNA, which suggests that circSPECC1 is nonlinear (Fig. 2C).

Following actinomycin D (Act D) treatment, circSPECC1 mRNA remained more stable than SPECC1 mRNA (Fig. 2D). The divergent primers could specifically amplify circSPECC1, whereas the convergent primers could amplify SPECC1 mRNA. Agarose gel electrophoresis further confirmed that circSPECC1 was resistant to RNase R degradation and was highly stable (Fig. 2E). Afterward, we performed cell component extraction and RNA FISH analysis in GBM cell lines (LN229 and U251) and corresponding TMZ-resistant GBM cell lines (LN229R and U251R), which showed that circSPECC1 was distributed in both the cytoplasm and nucleus in these cells, with a predominance in the cytoplasm (Fig. 2F, G). Overall, these findings demonstrated that circSPECC1 is a circular and stable transcript in GBM cell lines.

### CircSPECC1 suppressed the proliferation, migration, invasion, and colony formation abilities of GBM cells and restored GBM sensitivity to TMZ

**Fig. 3:**
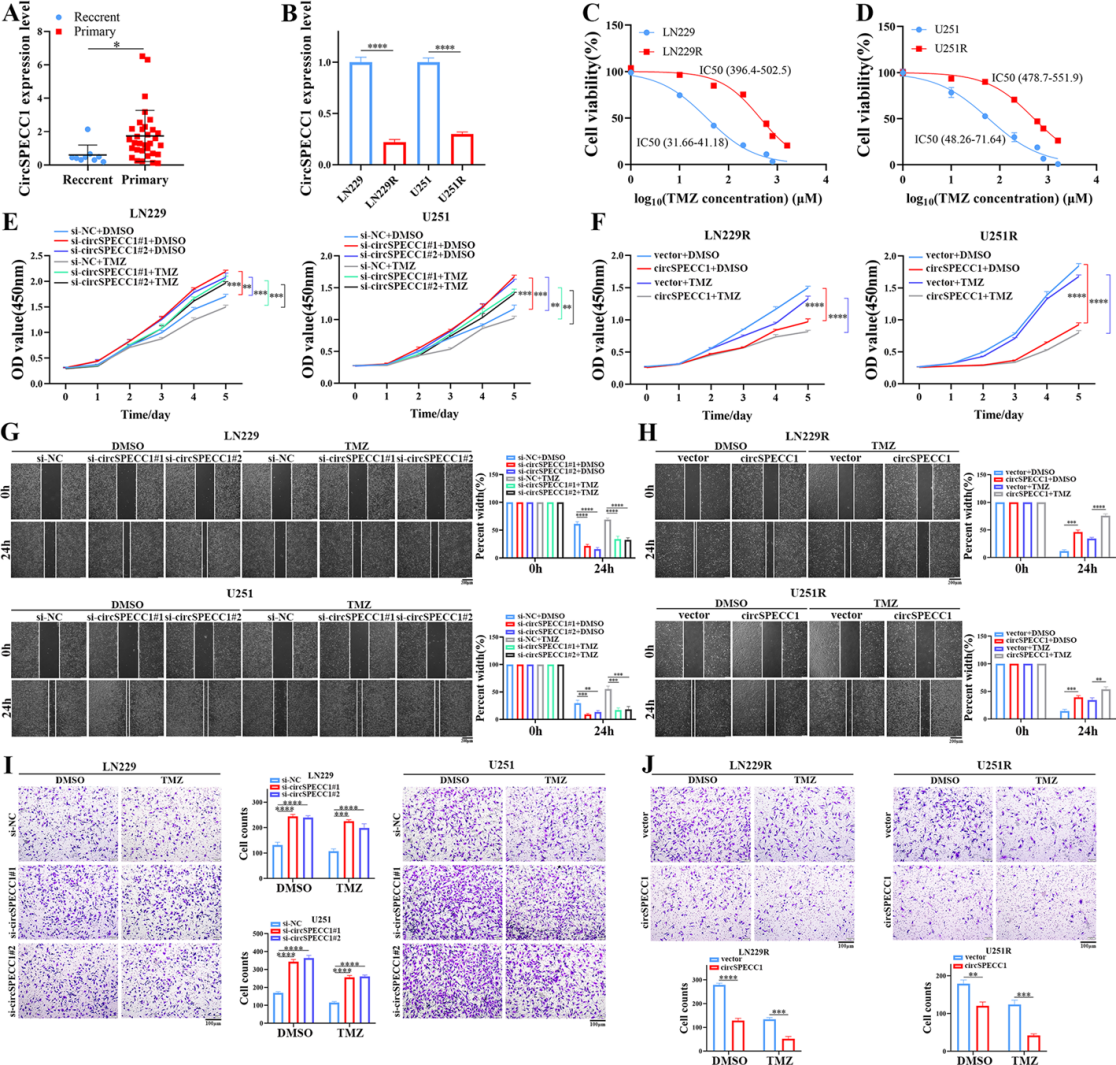
CircSPECC1 inhibited GBM and TMZ-resistant GBM cell lines proliferation, migration, and invasion abilities in vitro. **A** Analysis of circSPECC1 expression levels in primary (*n* = 32) and recurrent (*n* = 9) GBM samples. **B** Analysis of circSPECC1 expression in GBM cell lines (LN229 and U251) and corresponding TMZ-resistant GBM cell lines (LN229R and U251R). **C** Cell viability assay of LN229/LN229R cells treated with various concentrations of TMZ for 48 h. **D** Cell viability assay of U251/U251R cells treated with various concentrations of TMZ for 48 h. **E** CCK-8 assays for LN229 and U251 cells proliferation with circSPECC1 knockdown after dimethylsulfoxide (DMSO) or temozolomide (TMZ) co-treatment. **F** CCK-8 assays for LN229R and U251R cells proliferation with circSPECC1 overexpression after DMSO or TMZ co-treatment. **G** Wound healing assays for LN229 and U251 cells migration with circSPECC1 knockdown after DMSO or TMZ co-treatment. **H** Wound healing assays for LN229R and U251R cells migration with circSPECC1 overexpression after DMSO or TMZ co-treatment. **I** Transwell assays for LN229 and U251 cells invasion with circSPECC1 knockdown after DMSO or TMZ co-treatment. **J** Transwell assays for LN229R and U251R cells invasion with circSPECC1 overexpression after DMSO or TMZ co-treatment. *, *P* < 0.05; **, *P* < 0.01; ***, *P* < 0.001; ****, P < 0.0001

To investigate circSPECC1 expression levels in clinical samples, we analyzed circSPECC1 expression in nine samples from recurrent GBM patients after receiving standardized therapy and 32 samples from patients with primary GBM. The results showed that circSPECC1 expression in recurrent GBM tissues was lower than that in primary GBM tissues (Fig. 3A). Furthermore, circSPECC1 expression in LN229R cells was lower than that in LN229 cells. Similarly, the circSPECC1 expression level in U251R cells was lower than that in U251 cells (Fig. 3B). These findings demonstrated that circSPECC1 may function as a tumor suppressor gene in GBM and is associated with TMZ sensitivity.

To explore the biological functions of circSPECC1, we used two siRNAs targeting the junction site of circSPECC1 to knock down circSPECC1 expression in GBM cell lines (LN229 and U251), as well as a circSPECC1 overexpression plasmid to overexpress circSPECC1 in TMZ-resistant GBM cell lines (LN229R and U251R). The knockdown efficiency of circSPECC1 siRNAs and the overexpression efficiency of the circSPECC1 overexpression plasmid were verified via RT‒qPCR (Supplementary Fig. 1A‒B).

**Fig. 4:**
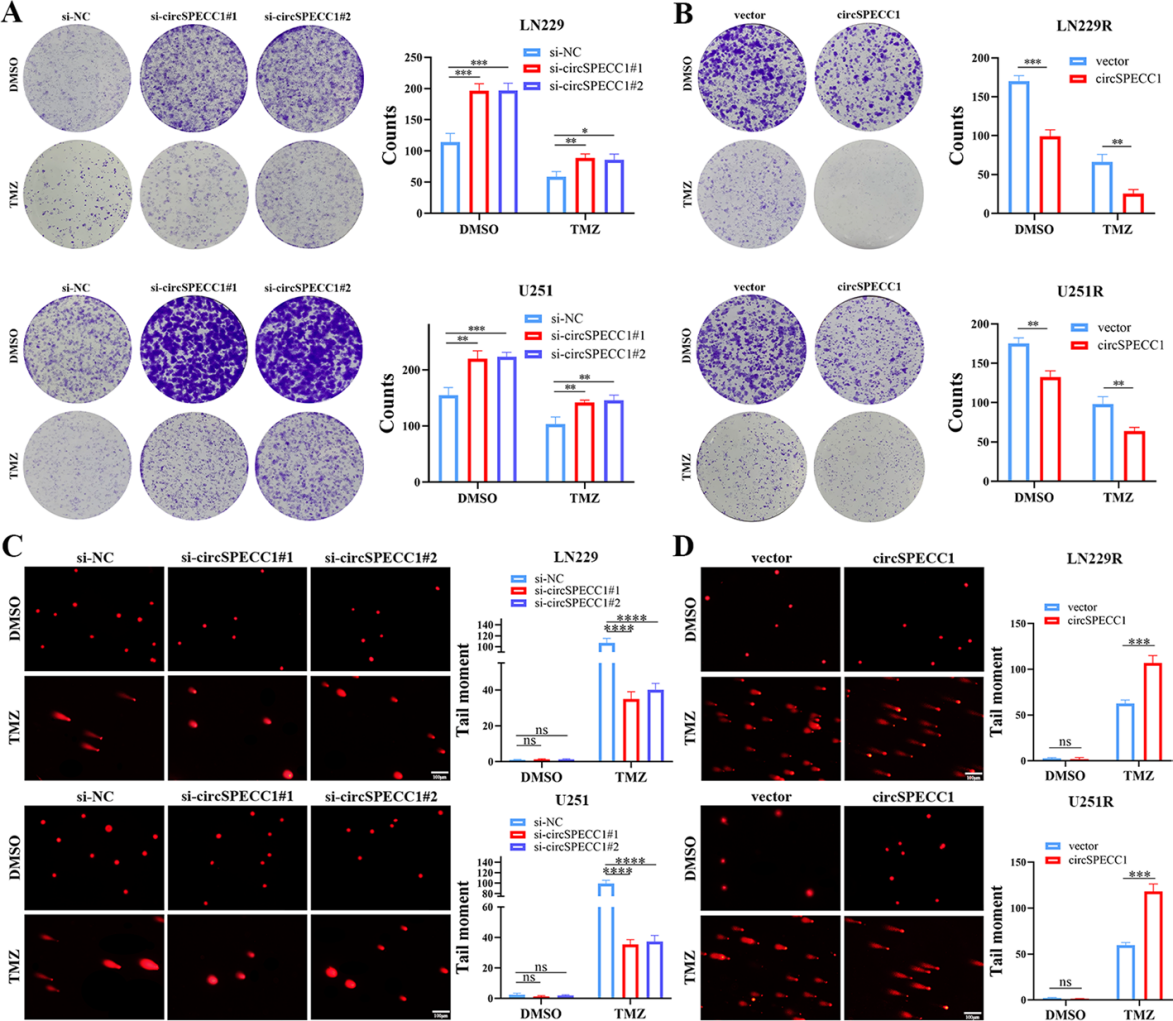
Colony formation and comet assays with circSPECC1 knockdown or overexpression in vitro. **A** Colony formation assays for LN229 and U251 cells colony formation ability with circSPECC1 knockdown after DMSO or TMZ co-treatment. **B** Colony formation assays for LN229R and U251R cells colony formation ability with circSPECC1 overexpression after DMSO or TMZ co-treatment. **C** Comet assays for LN229 and U251 cells DNA damage degree with circSPECC1 knockdown after DMSO or TMZ co-treatment. **D** Comet assays for LN229R and U251R cells DNA damage degree with circSPECC1 overexpression after DMSO or TMZ co-treatment. ns, *P* > 0.05; *, *P* < 0.05; **, *P* < 0.01; ***, *P* < 0.001; ****, *P* < 0.0001

Through CCK-8 assays, we obtained the half-maximal inhibitory concentration (IC_50_) values of TMZ in TMZ-resistant GBM cell lines (LN229R and U251R) and GBM cell lines (LN229 and U251). The results demonstrated that the IC_50_ values of GBM cell lines (LN229 and U251) were significantly lower than those of TMZ-resistant GBM cell lines (LN229R and U251R), thus indicating that TMZ-resistant GBM cell lines developed resistance to TMZ (Fig. 3C, D)_._ Furthermore, the CCK-8 assay results demonstrated that knockdown of circSPECC1 increased the proliferation of U251 and LN229 cells (Fig. 3E), and circSPECC1 overexpression inhibited the proliferation of LN229R and U251R cells (Fig. 3F), regardless of whether DMSO or TMZ was used for co-treatment. A wound healing assay showed that knockdown of circSPECC1 expression resulted in increased migration of both the LN229 and U251 cell lines (Fig. 3G), and overexpression of circSPECC1 inhibited the migration of the LN229R and U251R cell lines (Fig. 3H) after treatment with either DMSO or TMZ. Transwell experiments demonstrated that knockdown of circSPECC1 expression increased the invasion ability of the LN229 and U251 cell lines (Fig. 3I), and the overexpression of circSPECC1 inhibited the invasion ability of the LN229R and U251R cell lines (Fig. 3J), regardless of DMSO or TMZ treatment. Knockdown of circSPECC1 expression also led to an increase in the colony formation ability of the LN229 and U251 cell lines (Fig. 4A). The overexpression of circSPECC1 inhibited the colony formation ability of LN229R and U251R cells treated with either DMSO or TMZ (Fig. 4B).

Due to the difference in circSPECC1 expression between GBM cell lines and TMZ-resistant GBM cell lines, the impact of circSPECC1 expression on the TMZ sensitivity of GBM cells was verified by using an IC50 assay, a γH2AX immunofluorescence assay and a comet assay. The IC_50_ significantly increased when circSPECC1 was knocked down in GBM cell lines (LN229 and U251) and significantly decreased when circSPECC1 was overexpressed in resistant cell lines (LN229R and U251R) (Supplementary Fig. 2A, B)_._ The results of the γH2AX immunofluorescence assay indicated that there was no discernible variation in the intensity of γH2AX fluorescence in LN229 or U251 cells after knocking down circSPECC1 expression under DMSO treatment, whereas knocking down circSPECC1 expression under TMZ treatment increased the DNA damage repair ability of LN229 and U251 cells and decreased their sensitivity to TMZ (Supplementary Fig. 2C, D). The γH2AX fluorescence intensity in LN229R and U251R cells did not significantly differ from one another after overexpression of circSPECC1 under DMSO treatment, whereas the overexpression of circSPECC1 decreased the DNA damage repair ability of LN229R and U251R cells under TMZ treatment. In addition, the sensitivity of LN229R and U251R cells to TMZ was restored (Supplementary Fig. 3A, B). Moreover, the comet assay results demonstrated that there was no significant difference in the degree of DNA tailing in LN229 or U251 cells after knocking down circSPECC1 expression under DMSO treatment, whereas the knockdown of circSPECC1 resulted in a significant reduction in the degree of DNA tailing in LN229 or U251 cells after TMZ treatment. The findings demonstrated that the sensitivity of LN229 and U251 cells to TMZ was markedly decreased after circSPECC1 knockdown (Fig. 4C). In LN229R and U251R cells, there was no significant difference in the degree of DNA tailing after overexpressing circSPECC1 under DMSO treatment, whereas overexpression of circSPECC1 caused a significant increase in the degree of DNA tailing in LN229R and U251R cells after TMZ treatment (Fig. 4D). These findings demonstrated that circSPECC1 overexpression markedly increased the sensitivity of LN229R and U251R cells to TMZ.

These results showed that circSPECC1 functions as a tumor suppressor gene and inhibits the proliferation, migration, invasion and colony formation ability of GBM cells; additionally, the sensitivity of TMZ-resistant GBM cells to TMZ can be restored by circSPECC1.

### CircSPECC1 encoded the protein SPECC1-415aa

**Fig. 5:**
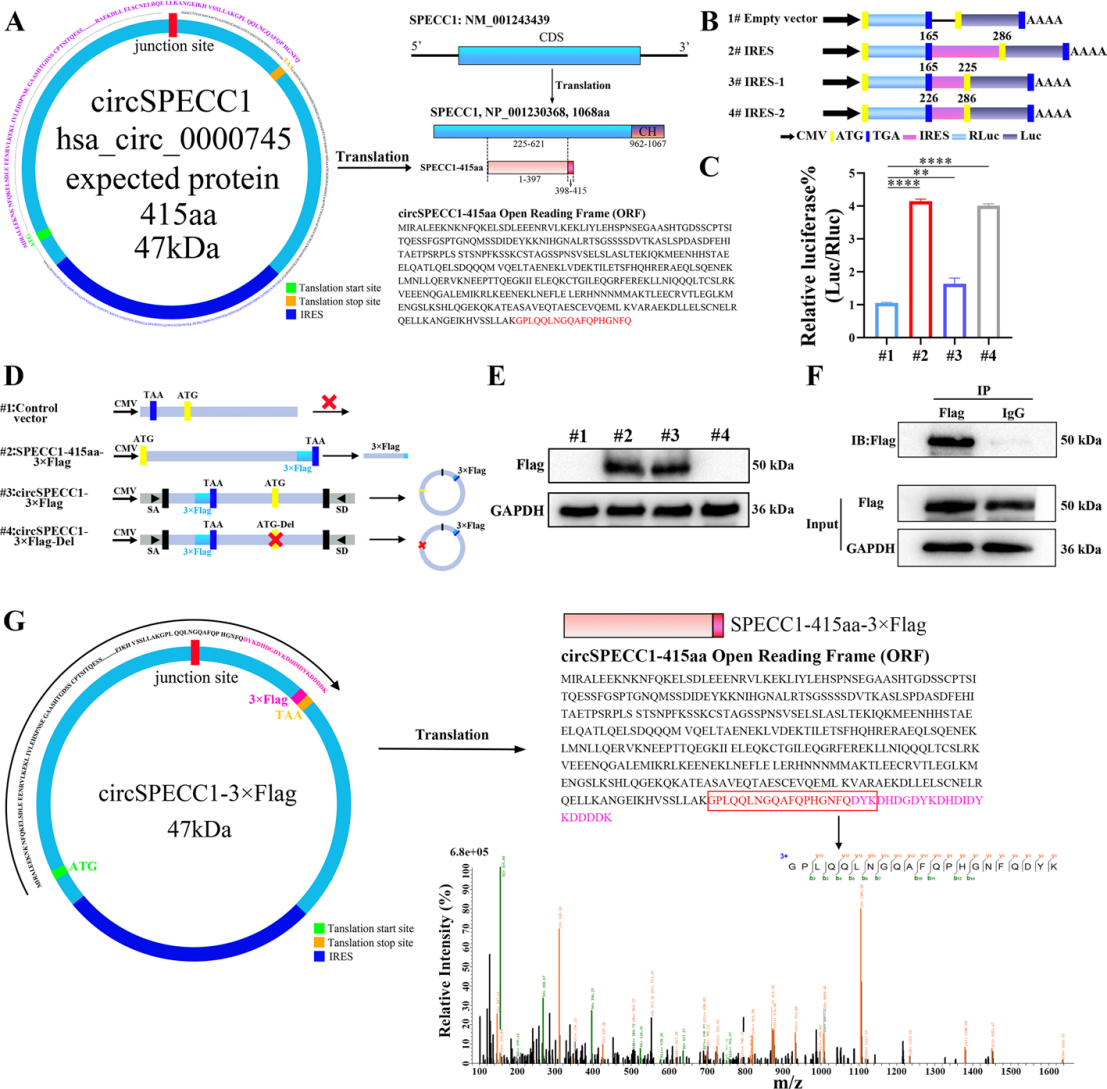
Identification of circSPECC1 coding capacity. **A** Diagram of circSPECC1 structure and explanation of amino acid alignment between the encoded new protein by circSPECC1 and the parent SPECC1 protein. **B** Full-length IRES sequences of circSPECC1 or IRES sequences of its different truncated mutants were cloned between Rluc/Luc reporters with independent start (ATG) and stop (TGA) codons, including full-length IRES (165-286nt), IRES-1 (165-225nt) and IRES-2 (225-286nt). **C** The relative luciferase activity of Luc/Rluc in empty vector, full-length IRES, IRES-1 and IRES-2 vector detected by dual luciferase assay. **D** Description of vectors used to detect whether circSPECC1 can encode the protein. Control vector: the flanking sequences were deleted and circSPECC1 could not be expressed. SPECC1-415aa-3 × Flag vector: a linearized ORF of circSPECC1 with a 3 × Flag tag was cloned into a linear plasmid and used as a positive control. CircSPECC1-3 × Flag vector: this vector had the flanking sequences, splice acceptor (SA) and splice donor (SD) and could be able to express circSPECC1 and SPECC1-415aa with Flag tag protein. CircSPECC1-3 × Flag-Del vector: the start codon (ATG) was censored, which could express circSPECC1 but not translate SPECC1-415aa. All vectors had CMV promoters. **E** The expression of SPECC1-415aa in 293 T cells transfected with the above plasmids by western blot assay. **F** IP and western blot experiments verified that the circSPECC1-3 × Flag plasmid expressed the Flag tag protein in 293 T cells and was specifically enriched by Flag antibody. **G** Schematic diagram of the new protein SPECC1-415aa translated by circSPECC1 and the secondary spectrum of the specific peptide of SPECC1-415aa-3 × Flag protein after IP-MS. **, *P* < 0.01; ****, *P* < 0.0001

The abovementioned studies confirmed that circSPECC1 has an IRES sequence and that it has an active structure that drives translation (Fig. 1E). Western blot experiments also showed that circSPECC1 could encode proteins (Fig. 1F). Subsequently, we further explored whether circSPECC1 can encode a protein in GBM. Based on the prediction information from circBase and TransCirc online, the open reading frame (ORF) of circSPECC1 translated a new protein known as SPECC1-415aa, with 415 amino acids and a molecular weight of approximately 47 kDa, as well as 18 specific amino acids at the end (GPLQQLNGQAFQPHGNFQ). The amino acid sequence 1–397 of SPECC1-415aa completely overlapped with the amino acid sequence 225–621 of the protein encoded by the parent SPECC1 mRNA, and the amino acid sequence 398–415 was specific for SPECC1-415aa (Fig. 5A). This observation suggests that this protein has a unique biological function.

Studies have shown that the IRES of circRNA contains two key regulatory elements, including the complementary sequence of 18S rRNA and the SuRE element (40–60 nt), which can promote the translation of circRNA in a cap-independent manner[23]. The ability of the IRES of circSPECC1 to drive its encoded protein and to encode the new protein SPECC1-415aa was verified. First, the IRES sequence activity of circSPECC1 was detected. Dual luciferase plasmids containing the full-length (165–286 nt) and truncated IRES mutant 1 (IRES-1, 165–225 nt) IRESs, as well as the truncated IRES mutant 2 (IRES-2, 226–286 nt) IRESs, were constructed to detect IRES-driven translation (Fig. 5B). The dual luciferase assay results showed that the full-length IRES (nt 165–286) sequence of circSPECC1 drove translation, and the IRES activity was mainly localized in the IRES-2 (nt 226–286) region, which represents the second half of the IRES (Fig. 5C). To further verify that the ORF of circSPECC1 could be translated into the SPECC1-415aa protein driven by the IRES sequence, we constructed a linear cDNA plasmid encoding SPECC1-415aa with a 3 × Flag tag sequence (p-SPECC1-415aa-Flag), a circSPECC1 overexpression plasmid (p-circSPECC1-Flag), and a circSPECC1 plasmid with an ATG deletion at the start codon (p-circSPECC1-Del-Flag) (Fig. 5D). Western blot results demonstrated that both the p-SPECC1-415aa-Flag and p-circSPECC1-Flag plasmids could express the predicted size protein (SPECC1-415aa). In contrast, the p-circSPECC1-Del-Flag plasmid without ATG at the start codon failed to express the protein of the predicted size (Fig. 5E). This indicated that the ORF of circSPECC1 was able to be translated into the protein SPECC1-415aa in the presence of the ATG initiation codon. These results indicated that the IRES sequence of circSPECC1 was active in driving translation and encoded a protein (SPECC1-415aa) with a molecular weight of approximately 47 kDa (approximately 50 kDa when the 3 × Flag tag was included).

Furthermore, the abovementioned assays confirmed that circSPECC1 could encode a protein and further confirmed that the protein encoded by circSPECC1 was consistent with the predicted protein amino acid sequence. 293 T cells were transfected with the constructed p-circSPECC1-Flag plasmid, after which immunoprecipitation and mass spectrometry amino acid sequence analysis (IP-MS) were conducted. Immunoprecipitation (IP) demonstrated that the p-circSPECC1-Flag plasmid was translated and expressed a protein with a molecular weight of approximately 50 kDa in 293 T cells, thus indicating that the SPECC1-415aa protein could be successfully expressed in 293 T cells and was highly enriched by the Flag tag antibody (Fig. 5F). The IP products enriched by the Flag tag antibody were analyzed by using mass spectrometry (MS). MS demonstrated a specific peptide segment unique to SPECC1-415aa (the red marked part represents the specific peptide sequence of SPECC1-415aa, and the purple marked part represents the 3 × Flag sequence) (Fig. 5G). The translation of the protein SPECC1-415aa was confirmed in circSPECC1.

### CircSPECC1 regulated GBM biological function and sensitivity to TMZ by encoding the protein SPECC1-415aa

**Fig. 6:**
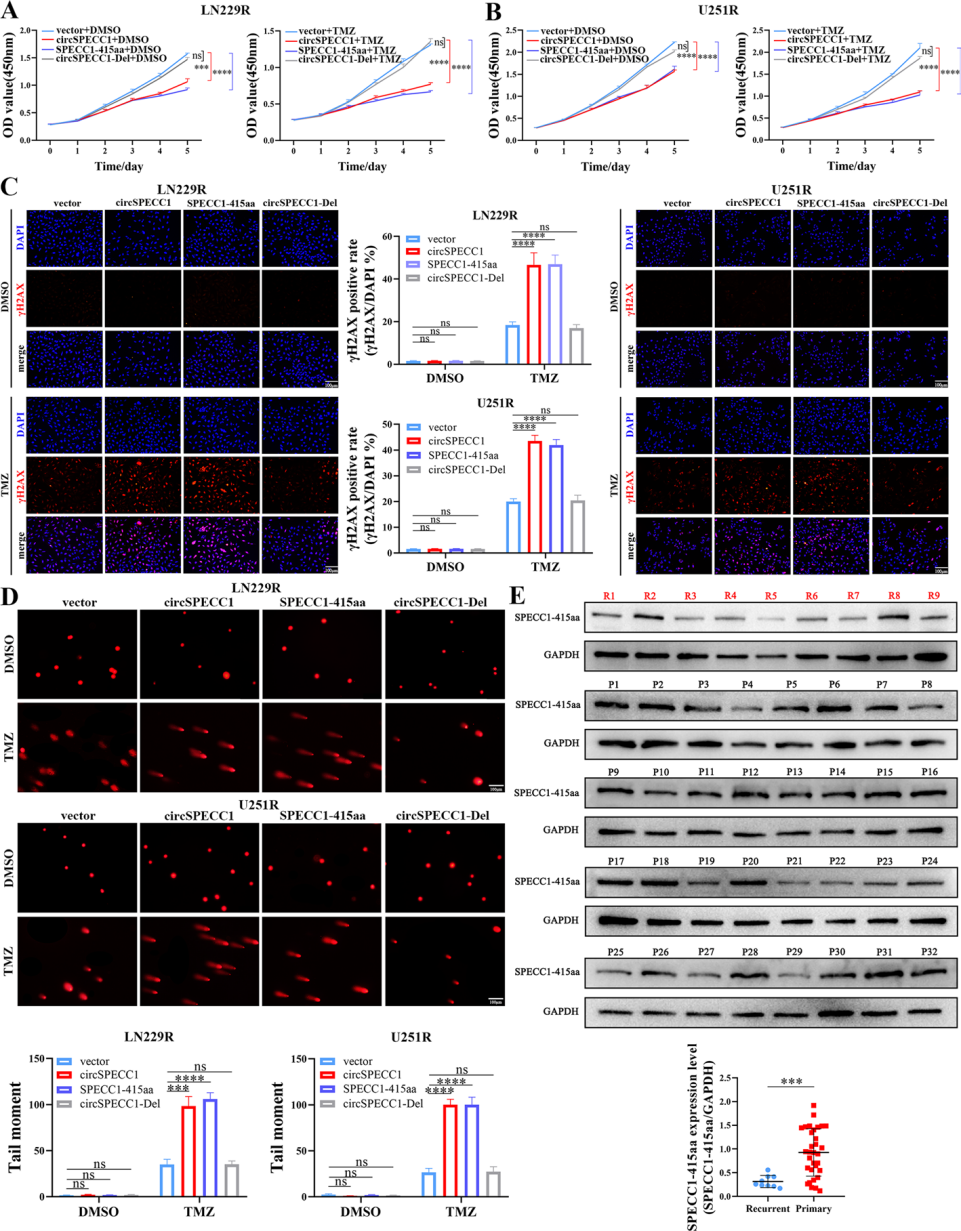
CircSPECC1 regulated the sensitivity of GBM to TMZ through SPECC1-415aa protein. **A** The effects of p-circSPECC1, p-SPECC1-415aa or p-circSPECC1-Del plasmids transfection on the proliferation ability of LN229R cells in the company of DMSO or TMZ. **B** The effects of p-circSPECC1, p-SPECC1-415aa or p-circSPECC1-Del plasmids transfection on the proliferation ability of U251R cells in the company of DMSO or TMZ. **C** The effects of p-circSPECC1, p-SPECC1-415aa or p-circSPECC1-Del plasmids transfection on the degree of DNA damage of LN229R and U251R cells by immunofluorescence assay in the presence of DMSO or TMZ. **D** In the presence of DMSO or TMZ, the effects of p-circSPECC1, p-SPECC1-415aa or p-circSPECC1-Del plasmids transfection on the degree of DNA damage in LN229R and U251R cells by comet assay. **E** The expression level of SPECC1-415aa protein in 9 samples of recurrent GBM patients and 32 samples of primary GBM patients via western blot experiment. ns, *P* > 0.05; ***, *P* < 0.001; ****, *P* < 0.0001

In contrast to primary GBM samples, recurrent GBM samples had aberrant expression of circSPECC1, thus regulating GBM cell sensitivity to TMZ. We hypothesized that circSPECC1 regulates GBM sensitivity to TMZ through its encoded protein SPECC1-415aa. First, CCK-8 assays demonstrated that the proliferation of LN229R and U251R cells transfected with p-circSPECC1 and p-SPECC1-415aa plasmids (both of which encode the SPECC1-415aa protein) was significantly inhibited by treatment with DMSO or TMZ. Cell proliferation was more inhibited in the TMZ cotreatment group than in the DMSO group. However, transfection of the p-circSPECC1-Del overexpression plasmid (which does not encode the SPECC1-415aa protein) did not significantly inhibit the proliferation of LN229R and U251R cells in the TMZ cotreatment group (Fig. 6A, B).

In addition, γH2AX immunofluorescence assays demonstrated that the extent of DNA damage did not significantly differ between LN229R and U251R cells transfected with p-circSPECC1, p-SPECC1-415aa or p-circSPECC1-Del plasmids under DMSO treatment. However, when cotreated with TMZ, the degree of DNA damage in LN229R and U251R cells transfected with p-circSPECC1 or p-SPECC1-415aa was significantly increased. Transfection of the p-circSPECC1-Del plasmid had no significant effect on the degree of DNA damage in LN229R and U251R cells treated with TMZ (Fig. 6C). Comet assays also demonstrated that DNA damage did not significantly differ between LN229R and U251R cells transfected with p-circSPECC1, p-SPECC1-415aa or p-circSPECC1-Del plasmids after DMSO treatment. However, compared with control cells, LN229R and U251R cells transfected with p-circSPECC1 and p-SPECC1-415aa plasmids showed a significant increase in comet tailing after TMZ treatment, indicating a significant increase in DNA damage. The overexpression of the p-circSPECC1-Del plasmid had no significant effect on DNA damage in LN229R or U251R cells (Fig. 6D). These results suggested that circSPECC1 regulated the DNA damage repair capacity of GBM to affect GBM sensitivity to TMZ by encoding the SPECC1-415aa protein.

The abovementioned results verified that the biological function of circSPECC1 in GBM was mainly achieved through its encoded protein SPECC1-415aa. We further analyzed the clinical characteristics of SPECC1-415aa. The mRNA expression level of circSPECC1 in primary and recurrent GBM tissues was consistent with the previous results, which demonstrated that the expression level of SPECC1-415aa in recurrent GBM tissues was significantly lower than that in primary GBM tissues (Fig. 6E).

### M^6^A modification promoted circSPECC1 expression

**Fig. 7:**
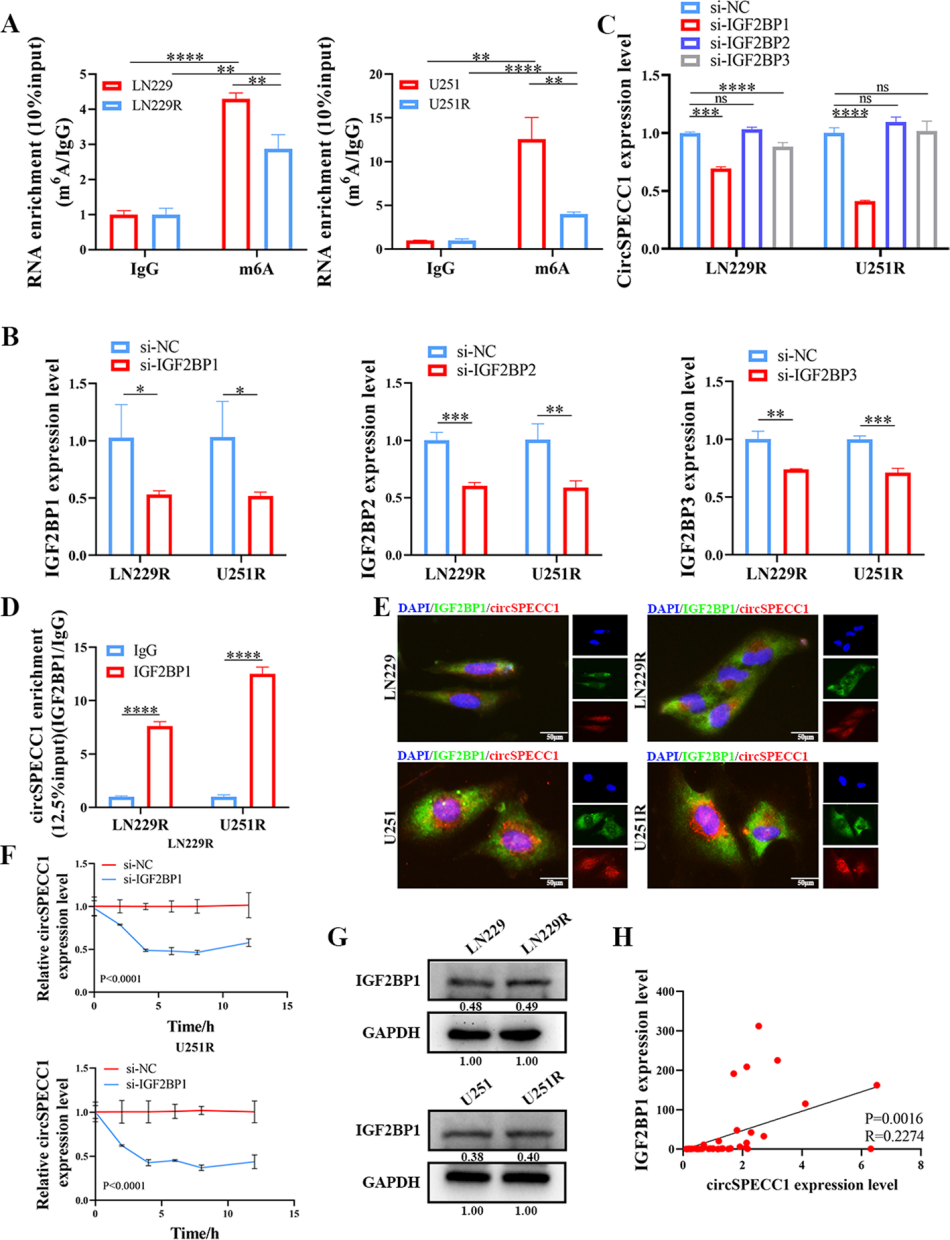
M^6^A modification on circSPECC1. **A** Verifying the m^6^A modification on circSPECC1 in TMZ-resistant GBM cell lines and GBM cell lines by MeRIP assay. **B** Verifying the knockdown efficiency of IGF2BP1/2/3 siRNA in LN229R and U251R cell lines. **C** RT-qPCR assay verifying the effect of IGF2BP1/2/3 knockdown on the expression level of circSPECC1 in LN229R and U251R cell lines. **D** RIP assay used to verify the binding of IGF2BP1 and circSPECC1 in LN229R and U251R cell lines. **E** Immunofluorescence assay used to verify the colocalization of IGF2BP1 and circSPECC1 in LN229, LN229R, U251 and U251R cells. **F** Effect of IGF2BP1 knockdown on the stability of circSPECC1 through Act D assay. **G** Western blot assay used to verify the expression level of IGF2BP1 between TMZ-resistant GBM cells and corresponding GBM cells. **H** Correlation analysis of IGF2BP1 mRNA and circSPECC1 expression in primary (*n* = 32) and recurrent (*n* = 9) GBM samples. ns, *P* > 0.05; *, *P* < 0.05; **, *P* < 0.01. ***, *P* < 0.001; ****, *P* < 0.0001

Our study demonstrated that circSPECC1 was expressed at low levels in recurrent GBM samples, and TransCirc online database prediction indicated the presence of m^6^A modification sites on circSPECC1. Therefore, we hypothesized that m^6^A modification may occur on circSPECC1 and affect its expression level in GBM. First, the predictive analysis of the SRAMP database demonstrated multiple m^6^A modification sites on circSPEEC1, including four sites with very high confidence (score > 0.8) (Supplementary Fig. 4). MeRIP assays demonstrated m^6^A modification on circSPECC1, and the level of m^6^A modification on circSPECC1 in a TMZ-resistant GBM cell line (LN229R/U251R) was lower than that in a GBM cell line (LN229/U251). These results indicated that the level of m^6^A modification on circSPECC1 was lower in TMZ-resistant GBM cell lines than in GBM cell lines (Fig. 7A).

Recent studies have shown that m^6^A reader proteins, including YTHDC1/2, YTHDF1/2/3, and IGF2BP1/2/3, can increase or reduce RNA stability [24–27]. Studies have shown that YTHDF and YTHDC family members mainly play biological roles in promoting circRNA degradation [28, 29]. However, IGF2BP family members mainly play biological roles in promoting circRNA stabilization [30, 31]. For example, circARHGAP12 was shown to contribute to the development of cervical cancer through the IGF2BP2/FOXM1 pathway, which is dependent on m^6^A. Moreover, RNA immunoprecipitation (RIP), fluorescence in-situ hybridization (FISH), and Act D experiments confirmed the binding of IGF2BP2 to circARHGAP12 and the ability of IGF2BP2 to increase the stability of circARHGAP12 [26]. Owing to the fact that the IGF2BP family can promote the stability of circRNAs, which is consistent with our meRIP experimental findings, IGF2BP1/2/3 were selected for further assessments.

To investigate the impact of IGF2BP1/2/3 on the stability of circSPECC1, siRNAs were used to knockdown the expression of IGF2BP1, IGF2BP2, and IGF2BP3. RT‒qPCR was used to assess the knockdown efficiency of si-IGF2BP1, si-IGF2BP2, and si-IGF2BP3 siRNAs in LN229R and U251R TMZ-resistant GBM cell lines. The results showed that the si-IGF2BP1, si-IGF2BP2, and si-IGF2BP3 siRNA sequences were effective (Fig. 7B). Afterwards, RT‒qPCR was used to further determine the effect of IGF2BP family members on circSPECC1 expression. The findings demonstrated that only IGF2BP1 affected the expression level of circSPECC1 after IGF2BP1/2/3 knockdown in the LN229R and U251R cell lines (Fig. 7C). CircSPECC1 expression was consistent with the trend of IGF2BP1 expression. Therefore, it was speculated that IGF2BP1 may be a m^6^A reader protein that binds to circSPECC1. Further RIP assays showed that IGF2BP1 could bind to circSPECC1 in the LN229R and U251R cell lines (Fig. 7D). Immunofluorescence experiments also demonstrated the colocalization of IGF2BP1 and circSPECC1 in LN229, LN229R, U251, and U251R cells, thus indicating that IGF2BP1 binds to circSPECC1 (Fig. 7E). Moreover, the Act D assay demonstrated that knockdown of IGF2BP1 expression in the LN229R and U251R cell lines resulted in a shortened half-life and decreased stability of circSPECC1 (Fig. 7F).

The level of m^6^A modification of circSPECC1 was lower in TMZ-resistant GBM cells than in corresponding GBM cells (Fig. 7A) and the m^6^A reader IGF2BP1 affected the stability of circSPECC1 (Fig. 7F). On the other hand, the protein expression level of IGF2BP1 was not significantly different between TMZ-resistant GBM cells and corresponding GBM cells (Fig. 7G). The above results suggested that the reduction of circSPECC1 expression in TMZ-resistant GBM cells compared with corresponding GBM cells was associated with the reduction of m^6^A modification occurring on circSPECC1. These findings indicated that the m^6^A reader protein IGF2BP1 bound to circSPECC1 and promoted its stability, which could also explain why circSPECC1 expression was reduced in TMZ-resistant GBM cells.

To validate the abovementioned findings in clinical samples, we further analyzed the correlation between circSPECC1 and IGF2BP1 mRNA in 9 recurrent GBM samples and 32 primary GBM samples. The results of the correlation analysis demonstrated a positive correlation between the levels of IGF2BP1 mRNA and circSPECC1 expression (Fig. 7H).

### SPECC1-415aa competitively bound ANXA2 with EGFR

Previous studies have verified that circSPECC1 functions as a tumor suppressor gene in GBM by encoding the protein SPECC1-415aa, and SPECC1-415aa can affect the sensitivity of GBM cells to TMZ. Afterward, the molecular mechanism by which SPECC1-415aa regulates TMZ sensitivity in GBM cells was explored. First, transcriptome sequencing was performed in the LN229 GBM cell line after knocking down circSPECC1. The cluster heatmap and volcano map showed the differentially expressed mRNAs after knocking down circSPECC1, and a total of 335 significantly downregulated mRNAs and 196 significantly upregulated mRNAs were identified (Supplementary Fig. 5A, B). Gene Ontology (GO) and Kyoto Encyclopedia of Genes and Genomes (KEGG) enrichment analyses were performed on the 531 differentially expressed mRNAs. KEGG enrichment analysis demonstrated that circSPECC1 mainly affected the PI3K-AKT signaling pathway, NF-kappa B signaling pathway, NOD-like receptor signaling pathway, IL-17 signaling pathway and ECM-receptor interaction (Supplementary Fig. 5C). In the GO analysis, biological process (BP) enrichment analysis showed that circSPECC1 mainly affected the negative regulation of cellular processes, response to stress, the cell surface receptor signaling pathway, regulation of apoptotic processes, regulation of cell death, and response to biotic stimuli (Supplementary Fig. 5D). Cellular component (CC) analysis demonstrated that the functional sites of circSPECC1-regulated genes were mainly localized in the cytosol, extracellular region, and extracellular matrix (Supplementary Fig. 5E). In addition, molecular function (MF) analysis demonstrated that the main molecular functions impacted by circSPECC1 included receptor regulation activity, signaling receptor binding, G protein-coupled receptor binding and receptor ligand activity (Supplementary Fig. 5F). These results suggested that circSPECC1 mainly affects the PI3K-AKT and NF-kappa B signaling pathways and mainly regulates biological processes, such as cell apoptosis, receptor ligand activity, signal receptor binding, and receptor regulatory activity, by regulating related mRNAs in the cytoplasm and extracellular matrix.

**Fig. 8:**
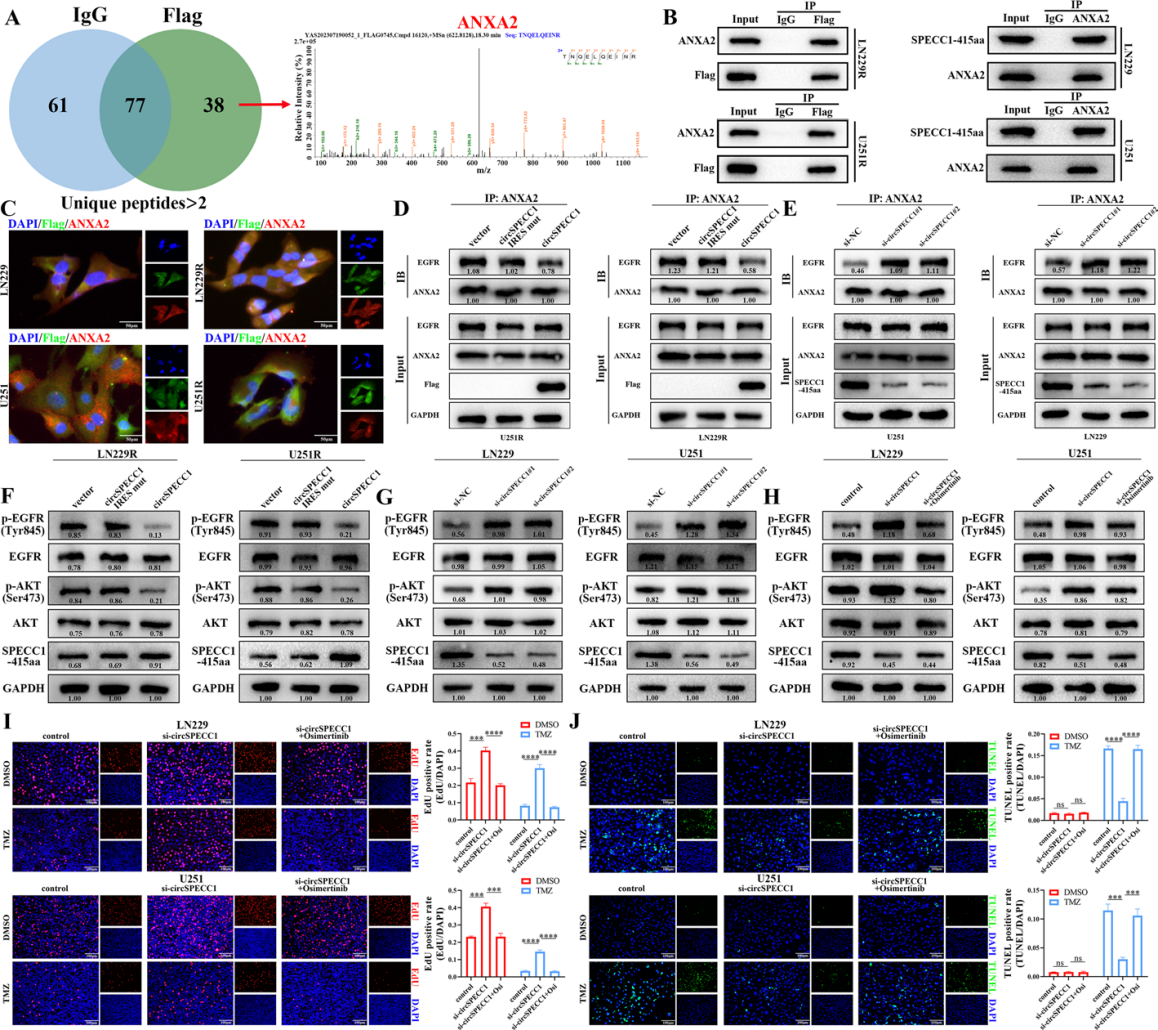
Validation of SPECC1-415aa binding to ANXA2 to regulate the EGFR-AKT pathway. **A** The binding proteins (Unique peptides > 2) of IgG group and Flag group and the secondary spectrum of ANXA2 specifically bound to the Flag protein in 293 T cells by IP-MS. **B** Verifying the bindings between endogenous ANXA2 protein and exogenous SPECC1-415aa-Flag (Left) in LN229R and U251R cells via IP assay. Verifying the bindings between endogenous ANXA2 protein and endogenous SPECC1-415aa (Right) in LN229 and U251 cellsby IP experiment. **C** Immunofluorescence assay verifying the interaction between ANXA2 protein and SPECC1-415aa protein in LN229, LN229R, U251 and U251R cell lines. **D** IP experiment verifying the binding level of ANXA2 and EGFR after the overexpression of circSPECC1 in U251R and LN229R cells. **E** IP experiment verifying the binding level of ANXA2 and EGFR after the circSPECC1 knockdown in U251 and LN229 cells. **F** Impact of circSPECC1 overexpression on activation of EGFR-AKT pathway in LN229R and U251R cells. **G** Effect of circSPECC1 knockdown on activation of EGFR-AKT pathway in LN229 and U251 cells. **H** Effect of circSPECC1 knockdown or circSPECC1 knockdown combined with EGFR blocker Osimertinib on the activation of EGFR-AKT pathway in LN229 and U251. **I** Osimertinib could restore the sensitivity of LN229 and U251 cells to TMZ after knocking down circSPECC1 via EdU experiment. **J** Osimertinib could restore the sensitivity of LN229 and U251 cells to TMZ after knocking down circSPECC1 via TUNEL experiment. ns, *P* > 0.05; ***, *P* < 0.001; ****, *P* < 0.0001

To investigate the molecular mechanism by which SPECC1-415aa regulates the sensitivity of GBM cells to TMZ, immunoprecipitation-mass spectrometry (IP-MS) experiments were performed in 293 T cells using a linear SPECC1-415aa overexpression plasmid with a 3 × Flag tag (p-SPECC1-415aa-Flag). After screening for the number of unique peptides (more than 2), the results showed that IgG and Flag had 77 common binding proteins, with 61 specific binding proteins in the IgG group and 38 specific binding proteins in the Flag group (Fig. 8A, left panel). Among these 38 proteins, we further screened proteins with more than five unique peptides, and a total of six proteins were found, including HSPA1A, MYH6, MYL3, CASP14, ANXA2, and ACTC1. Through comprehensive analysis of its subcellular localization and biological functions in tumors, as well as via enrichment analyses via transcriptome sequencing, annexin A2 (ANXA2) was identified for further study. Figure 8A (right panel) shows the secondary spectra of the specific peptide of ANXA2 that can bind to SPECC1-415aa in the IP‒MS assay. Further IP experiments showed that exogenous SPECC1-415aa-Flag was able to bind to the endogenous ANXA2 protein in the LN229R and U251R cell lines (Fig. 8B, left panel). The IP experiments further showed that endogenous SPECC1-415aa could bind to the endogenous ANXA2 protein in LN229 and U251 cell lines (Fig. 8B, right panel). Immunofluorescence assays also verified that ANXA2 colocalized with SPECC1-415aa in LN229, LN229R, U251, and U251R cells and mainly colocalized to the cytoplasm (Fig. 8C). These results indicated that SPECC1-415aa can bind to the ANXA2 protein in GBM cell lines and corresponding TMZ-resistant GBM cell lines.

Current studies have shown that proteins encoded by circRNAs can not only promote the binding of target proteins with their interacting proteins but also competitively inhibit the binding of target proteins with their interacting proteins to perform biological functions in tumors [32, 33]. For example, Wang et al. reported that circMAPK14-encoded MAPK14-175aa reduced the nuclear translocation of MAPK14 by competitively binding to MKK6, thereby promoting ubiquitin-mediated FOXC1 degradation [32]. Numerous studies have shown that ANXA2 functions as an oncogene in tumors and promotes chemotherapy resistance [34, 35]. Wang et al. reported that ANXA2 may stimulate AKT activation, promote tumor growth and metastasis and interact with TIM-4 to regulate oxidative phosphorylation in lung cancer cells via the ANXA2/PI3K/AKT/OPA1 pathway to accelerate tumor progression [36]. Our KEGG enrichment analysis of the transcriptome data also demonstrated that circSPECC1 functions as a tumor suppressor gene in GBM by primarily influencing the PI3K-AKT signaling pathway. In addition, P Chaudhary et al. reported that ANXA2 interacts with epidermal growth factor receptor (EGFR) on the cell surface and plays a crucial role in malignant phenotypes (such as cancer cell proliferation and migration) by modulating the activation of the Raf-MEK-ERK and PI3K-AKT pathways [37]. Therefore, we hypothesized that SPECC1-415aa inhibited the binding of ANXA2 to its target protein EGFR by competitively binding to ANXA2 to inhibit the phosphorylation of EGFR on the cell membrane and the activation of the PI3K-AKT signaling pathway. Moreover, the SPECC1-415aa/ANXA2/EGFR/AKT axis can suppress the malignant phenotypes of GBM cells and restore the sensitivity of TMZ-resistant GBM cells to TMZ. To verify this hypothesis, we constructed a p-circSPECC1-IRES mutant (mut) plasmid with an IRES sequence mutation, which prevented circSPECC1 from initiating translation. Through IP experiments, our findings demonstrated that the binding of ANXA2 to EGFR was reduced after circSPECC1 was overexpressed in the U251R and LN229R cell lines (Fig. 8D). The results of the vector group and circSPECC1 IRES mutation group were consistent. The overexpression of the circSPECC1 IRES mutation plasmid did not affect the binding of ANXA2 to EGFR. Only when circSPECC1 was overexpressed and the SPECC1-415aa protein was translated the binding of ANXA2 to EGFR reduced (Fig. 8D). In contrast, the knockdown of circSPECC1 expression in the U251 and LN229 cell lines increased the binding of ANXA2 to EGFR (Fig. 8E). These results suggested that SPECC1-415aa can bind to ANXA2 and competitively inhibit the binding of ANXA2 to its target protein EGFR.

### SPECC1-415aa interacted with ANXA2 to inhibit the phosphorylation of EGFR and AKT, thus resulting in increased sensitivity of GBM to TMZ

Studies have shown that EGFR Y845 transphosphorylation is related to its autophosphorylation and kinase activity [38]. EGFR is stimulated by ligands (such as EGF) to form homodimers or heterodimers with one of the other three family members, which correspondingly phosphorylate the intracellular domain of EGFR through Src kinase (transphosphorylation, Tyr845) or autophosphorylation. When phosphorylated EGFR binds to downstream adaptor proteins, it creates docking sites that trigger a variety of downstream signaling pathways, such as the AKT pathway [39, 40]. Vijaya Kumar Pidugu et al. discovered that p-EGFR endosomal recycling was improved by the interaction of IFIT1 and IFIT3 with ANXA2. Moreover, p-EGFR and p-AKT (Ser473) were downregulated in IFIT1- or IFIT3-overexpressing cells when ANXA2 was depleted by siRNA [41]. We further investigated whether the competitive inhibition of ANXA2 binding to its target EGFR by SPECC1-415aa affected the activation of p-EGFR (Tyr845) and p-AKT (Ser473). Western blot experiments demonstrated that the levels of p-EGFR (Tyr845) and p-AKT (Ser473) were significantly reduced after circSPECC1 overexpression in LN229R and U251R cells, and there was no discernible difference in the overall protein levels of EGFR and AKT (Fig. 8F). In addition, the levels of p-EGFR (Tyr845) and p-AKT (Ser473) were significantly increased after circSPECC1 knockdown in LN229 and U251 cells, and there was no discernible difference in the overall protein levels of EGFR and AKT (Fig. 8G). These results indicated that SPECC1-415aa competitively bound to ANXA2 and inhibited the binding of ANXA2 to its target protein EGFR, which correspondingly restrained the phosphorylation and activation of EGFR and its pathway protein AKT.

To further verify that SPECC1-415aa regulates the TMZ sensitivity of GBM cells through the downstream EGFR-AKT signaling pathway, recovery experiments were performed by using the EGFR blocker osimertinib. Western blot experiments demonstrated that circSPECC1 knockdown resulted in increased activation of p-EGFR and p-AKT in the LN229 and U251 cell lines (Fig. 8H). Blockade of EGFR activity with osimertinib restored the increased activation of p-EGFR and p-AKT that resulted from circSPECC1 knockdown (Fig. 8H). EdU experiments demonstrated that the knockdown of circSPECC1 in LN229 and U251 cells led to an increase in cell proliferation, and knockdown of circSPECC1 in conjunction with blockage of EGFR activity with osimertinib restored their proliferation ability to a similar level of the control group after DMSO treatment (Fig. 8I). In addition, knockdown of circSPECC1 in LN229 and U251 cells led to TMZ resistance and increased cell proliferation, and knockdown of circSPECC1 combined with blockade of EGFR activity with osimertinib restored cell sensitivity to TMZ after TMZ treatment, thus resulting in decreased proliferative capacity (Fig. 8I). TUNEL assays also showed that the knockdown of circSPECC1 or circSPECC1 combined with osimertinib had no significant effect on apoptosis in LN229 and U251 cells after treatment with DMSO. After TMZ treatment, knockdown of circSPECC1 in LN229 and U251 cells resulted in cell resistance to TMZ and decreased apoptosis, and knockdown of circSPECC1 combined with osimertinib restored cell sensitivity to TMZ, thus resulting in increased apoptosis (Fig. 8J). Furthermore, a γH2AX immunofluorescence assay demonstrated that circSPECC1 knockdown in LN229 and U251 cells led to TMZ resistance and reduced DNA damage, and knockdown of circSPECC1 combined with osimertinib restored the sensitivity of cells to TMZ after TMZ treatment, thus resulting in increased DNA damage (Fig. S6). These results suggested that SPECC1-415aa regulated the biological functions of GBM and GBM cell sensitivity to TMZ through the downstream ANXA2-EGFR-AKT signaling pathway.

### SPECC1-415aa reversed TMZ sensitivity in TMZ-resistant GBM cells in vivo

**Fig. 9:**
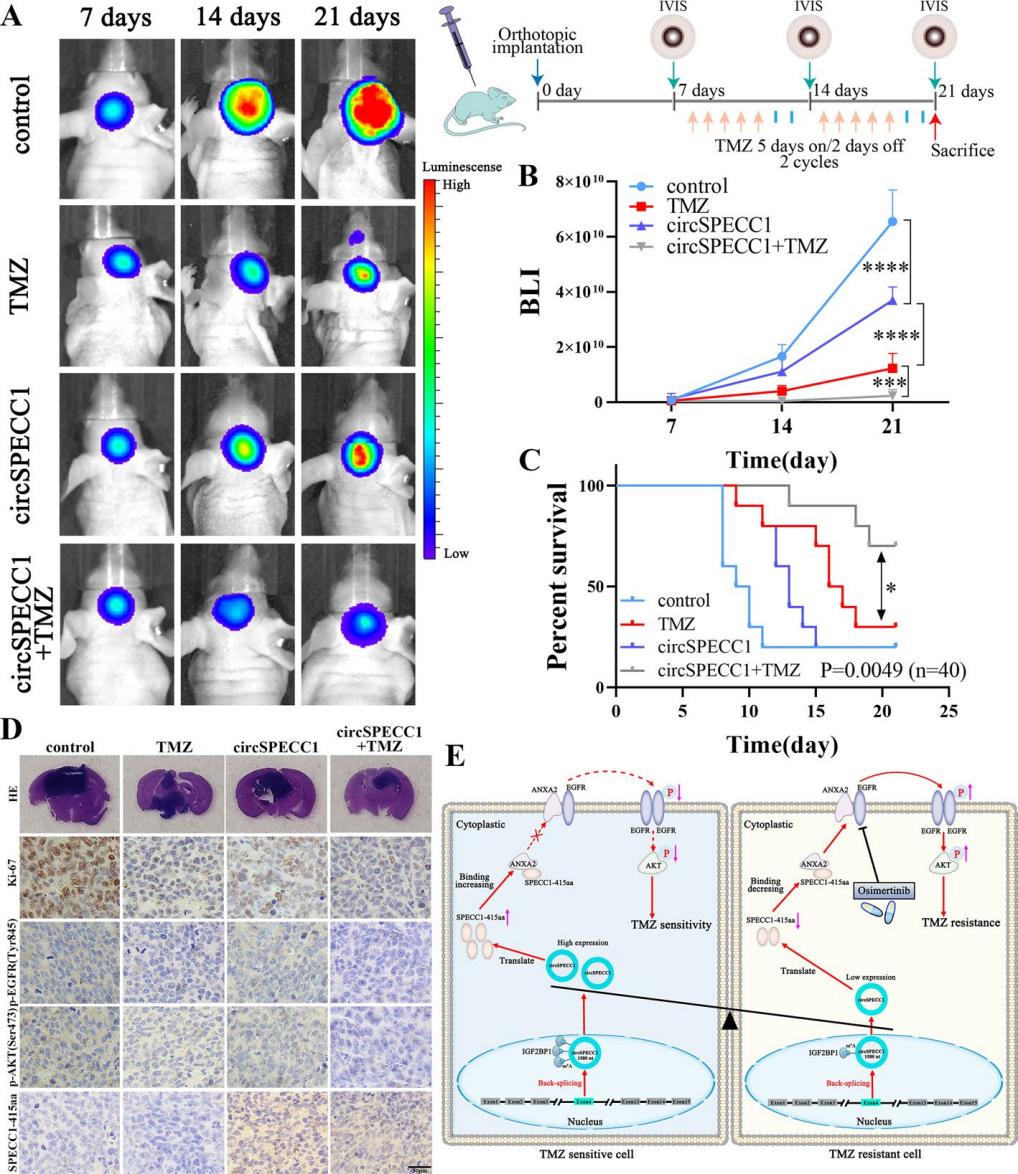
Overexpression of circSPECC1 restored TMZ sensitivity of TMZ-resistant GBM cells in vivo. **A** Schematic diagram of LN229R nude mice intracranial tumor inoculation and TMZ injection protocol (right panel). The IVIS bioluminescence images of the control, TMZ, circSPECC1 overexpression, and circSPECC1 overexpression combined with TMZ group at 7, 14, and 21 days after treatment (left panel). **B** Quantitative analysis of bioluminescence intensity of control, TMZ, circSPECC1 overexpression, and circSPECC1 overexpression combined with TMZ group after 7, 14, and 21 days of treatment. **C** Kaplan–Meier survival analysis of mice orthotopic transplanted with LN229R cells. **D** Representative H–E staining images of control, TMZ, circSPECC1 overexpression, and circSPECC1 overexpression combined with TMZ co-treatment group tissues. Representative immunohistochemical (IHC) images of control, TMZ, circSPECC1 overexpression, and circSPECC1 overexpression combined with TMZ co-treatment group tissues stained with Ki-67, p-EGFR (Tyr845), p-AKT (Ser473), and SPECC1-415aa. **E** Schematic model of the function of circSPECC1 in regulating GBM sensitivity to TMZ. *, *P* < 0.05; ***, *P* < 0.001; ****, *P* < 0.0001

It has been demonstrated that the overexpression of circSPECC1 can restore TMZ sensitivity in TMZ-resistant GBM cells in vitro. To further validate the abovementioned results in vivo, we constructed intracranial TMZ-resistant GBM xenograft models in nude mice, which were intracranially inoculated with LN229R-luc-control cell lines stably expressing luciferase or LN229R-luc-circSPECC1 cell lines stably expressing luciferase and circSPECC1. The TMZ treatment group received two cycles of TMZ treatment (continuous intraperitoneal injections for 5 days, stopped for 2 days as a cycle) on days 7 and 14 (Fig. 9A, right panel). Tumor size was assessed by using bioluminescence (BLI) intensity via IVIS small animal imaging at 7, 14, and 21 days. IVIS bioluminescence images and BLI photon number quantification showed that circSPECC1 overexpression alone inhibited tumor growth but was less effective than TMZ treatment alone, whereas TMZ combined with circSPECC1 overexpression increased tumor sensitivity to TMZ and significantly suppressed tumor growth (Fig. 9A, B). K‒M survival analysis also demonstrated that the survival time of nude mice treated with TMZ combined with circSPECC1 overexpression was the longest (median survival time > 21 days), which was significantly different from that of the TMZ treatment group (median survival time of 16.5 days) (Fig. 9C). Hematoxylin and eosin (HE) staining showed that the TMZ combined with circSPECC1 overexpression group had the smallest tumor volume compared with the other three groups, thus indicating that tumor growth was significantly inhibited (Fig. 9D). However, the tumor volume in the TMZ combined with circSPECC1 overexpression group was smaller than that in the TMZ treatment group and circSPECC1 overexpression group, which was consistent with the IVIS imaging results (Fig. 9D).

Immunohistochemical (IHC) staining demonstrated that Ki-67, p-EGFR (Tyr845), and p-AKT (Ser473) expression levels were highest in the control group and lowest in the circSPECC1 overexpression combined with TMZ treatment group; these levels were lower than those in the TMZ treatment group and circSPECC1 overexpression group (Fig. 9D). Immunohistochemical (IHC) staining also showed that SPECC1-415aa expression levels were higher in the circSPECC1 and circSPECC1 overexpression combined with TMZ treatment groups than that in the control and TMZ groups (Fig. 9D). The results showed that cell proliferation and EGFR-AKT signaling pathway activity were significantly inhibited in the circSPECC1 overexpression combined with TMZ treatment group, and circSPECC1 overexpression restored the sensitivity of TMZ-resistant GBM to TMZ (Fig. 9D). The abovementioned results indicated that circSPECC1 overexpression combined with TMZ can inhibit the growth of TMZ-resistant GBM (recurrent GBM) and can restore the sensitivity of TMZ-resistant GBM to TMZ.

## Discussion

Recent studies have shown that the occurrence and development of human tumors are significantly influenced by the dysregulation of circular RNA (circRNA) expression [6, 7]. Numerous studies have demonstrated that aberrantly expressed circRNAs are important in the development of glioblastoma (GBM) and can regulate its pathological process through different mechanisms [10, 11]. In addition, circRNAs can also encode proteins or peptides, thereby affecting the malignant phenotypes of GBM, such as proliferation, migration, invasion, and chemoradiotherapy resistance [19]. Patients with GBM are prone to developing resistance to TMZ during standard treatment, thus leading to recurrence. Therefore, it is crucial to continue researching the mechanism of circRNA and its encoded products in TMZ sensitivity and GBM recurrence. To investigate the possible role of circRNAs in GBM recurrence, we screened differentially expressed circRNAs with translational potential between primary and recurrent GBM samples via circRNAs microarray. The strict inclusion criteria led to the identification of circSPECC1 and its selection for additional research. CircSPECC1 was significantly downregulated in recurrent GBM compared with primary GBM (Fig. 3A). Our study demonstrated that circSPECC1 could inhibit GBM cell proliferation, migration, invasion, and colony formation and restore TMZ sensitivity in TMZ-resistant GBM cells (Fig. 3E–J and Fig. 4A, B). These findings suggested that circSPECC1 functions as a cancer suppressor gene in the occurrence and development of GBM.

Research has demonstrated that circRNAs function as transcriptional regulators to regulate host gene expression [16]. CircRNAs can also act as microRNA sponges and act as ceRNAs to regulate mRNA expression [42]. In addition, circRNAs are considered “noncoding” RNAs, which are “noise” generated during transcription [16]. However, a number of studies have shown that circRNAs can encode peptides or proteins [43, 44]. For example, Xiong et al. reported that the ubiquitin E3 ligase complex (CUL5-ASB6) can be recruited by the 121 amino acid protein circINSIG1-121aa (which is encoded by INSIG1) to enhance K48-linked ubiquitination at lysine 156 and 158, which are important regulators of cholesterol metabolism. This process induces cholesterol biosynthesis and promotes the growth and metastasis of CRC [43]. Peng et al. showed that circAXIN1 encodes a new protein of 295 amino acids, which is known as AXIN1-295aa. Axin1-295aa can compete with AXIN1 for binding to APC, thus resulting in the release and nuclear translocation of β-catenin. Nuclear translocation of β-catenin activates the Wnt pathway and induces Wnt-dependent gene expression, thus promoting the proliferation, invasion and migration of gastric cancer cells [44]. Our finding also showed that circSPECC1 can encode a new protein known as SPECC1-415aa, which can restrain the proliferation, invasion, migration, and colony formation abilities of GBM cells and restore the sensitivity of TMZ-resistant GBM cells to TMZ by encoding a new protein (Fig. 3E–J and Fig. 4A, B).

In eukaryotic cells, m^6^A modification is one of the most prevalent types of RNA chemical modifications, and m^6^A modification can occur on noncoding RNA (ncRNA) [45]. Numerous studies have demonstrated that m^6^A modification can also occur on circRNAs, and m^6^A-modified circRNAs have important functions in tumors [46]. Shao et al. showed that YTHDF2 can initiate the decay of m^6^A-containing circRNAs. The downregulation of circAFF2 in HCT-116 and HT-29 cells was reversed by YTHDF2 knockdown, thus suggesting that ALKBH5-mediated demethylation regulated circAFF2 expression and that YTHDF2 mediated circAFF2 degradation in a m^6^A-dependent manner [47]. Wang et al. demonstrated that circMPP1 directly binds to YTHDC1, and the low expression of YTHDC1 in the JEG3 choriocarcinoma cell line led to decreased degradation of circMPP1, which caused increased expression of circMPP1, thus leading to placental trophoblast dysfunction [29]. Ji et al. reported that circARHGAP12 contributes to the development of cervical cancer by acting as an oncogene via the IGF2BP2/FOXM1 pathway in a m^6^A-dependent manner. Furthermore, the binding of IGF2BP2 to circARHGAP12 was confirmed via RIP, FISH, and Act D experiments, and IGF2BP2 promoted the stability of circARHGAP12 [26]. In this study, m^6^A modification of circSPECC1 was detected, and the level of m^6^A modification in TMZ-resistant GBM cell lines was lower than that in the corresponding GBM cell lines (Fig. 7A). In addition, the m^6^A reader protein IGF2BP1 could bind to circSPECC1 and increase its expression and stability (Fig. 7D–F). This scenario could also explain the low expression level of circSPECC1 in recurrent GBM samples and TMZ-resistant GBM cell lines, and the low expression of circSPECC1 could lead to TMZ resistance in GBM cells.

To explore the molecular mechanism of circSPECC1, transcriptome sequencing was performed on GBM cell lines. The results showed that circSPECC1 mainly regulated PI3K-AKT and NF-kappa B signaling pathways (Supplementary Fig. 5C). Moreover, circSPECC1 mainly regulated biological processes such as cell apoptosis, signal receptor binding, receptor regulatory activity and receptor ligand activity by regulating related genes in the cytoplasm and extracellular matrix (Supplementary Fig. 5D). IP-MS data showed that the new protein SPECC1-415aa encoded by circSPECC1 could bind to ANXA2 (Fig. 8B). Current studies have shown that circRNA-encoded proteins can not only promote the binding of target proteins with their interacting proteins but also competitively inhibit the binding of target proteins with their interacting proteins to perform biological functions in tumors. Wang et al. reported that the MAPK14-175 aa region encoded by circMAPK14 reduced the nuclear translocation of MAPK14 by competitive binding to MKK6, thereby promoting ubiquitin-mediated FOXC1 degradation [32]. We hypothesized that SPECC1-415aa may bind to ANXA2 to promote or inhibit the binding of ANXA2 to its target proteins. Research has indicated that ANXA2 functions as an oncogene in tumors and can promote chemotherapy resistance [34, 35]. Through its interaction with EGFR on the cell surface, ANXA2 regulates the activation of EGFR and the downstream PI3K-AKT signaling pathway, which is important for malignant phenotypes, such as cancer cell proliferation and migration [36]. EGFR is a transmembrane receptor that can bind with ligands of members of the epidermal growth factor family (EGF-family). Binding of the ligands to EGFR leads to EGFR dimerization, which in turn leads to EGFR autophosphorylation and activation of downstream signaling pathways, including PI3K-AKT and NF-kappa B signaling pathways [48–50]. The activation of EGFR can trigger multiple intracellular signaling cascades that play an important role in regulating various cellular functions, including proliferation, motility, differentiation, and apoptosis [51, 52]. Our research demonstrated that SPECC1-415aa bound to ANXA2 and inhibited the binding of ANXA2 to its target protein EGFR, thereby inhibiting the phosphorylation of EGFR on the cell membrane and the activation of AKT signaling pathway proteins and leading to the suppression of the malignant phenotype and restoration of GBM cell sensitivity to TMZ (Fig. 8 D-J and Supplementary Fig. 6). Transcriptome results showed that circSPECC1 affected the PI3K-AKT and NF-kappa B signaling pathways and mainly regulated biological processes, such as cell stress, cell apoptosis, and cell death by regulating related genes. Therefore, we hypothesized that SPECC1-415aa/ANXA2 /EGFR/AKT was a key event in the regulation of the above genes (Fig. 9E). In the future, we will further study the molecular mechanism behind this regulation.

## Conclusions

CircSPECC1 was expressed at lower levels in recurrent GBM than in primary GBM and was regulated by the m^6^A reader protein IGF2BP1. CircSPECC1 encodes a new protein known as SPECC1-415aa, which can competitively bind to ANXA2, thus blocking the binding of ANXA2 to EGFR and inhibiting the phosphorylation of EGFR and AKT and thereby restoring the sensitivity of TMZ-resistant GBM cells to TMZ. These results suggest that circSPECC1 can be used as a potential molecular target to inhibit recurrent GBM growth, improve TMZ resistance in TMZ-resistant GBM and prevent GBM recurrence.

## Supplementary Information

Supplementary Material 1.

Supplementary Material 2.

## Data Availability

Data available from the authors upon reasonable request.

## Abbreviations

CircRNA: Circular RNA
GBM: Glioblastoma
WHO: World Health Organization
TMZ: Temozolomide
IRES: Internal ribosomal entry site
NTT: Nontumor brain tissue
HGG: High-grade glioma
SPECC1: Sperm antigen with calponin homology and coiled-coil domains 1
Act D: Actinomycin D
IP-MS: Immunoprecipitation and mass spectrometry amino acid sequence analysis
IP: Immunoprecipitation
GO: Gene Ontology
KEGG: Kyoto Encyclopedia of Genes and Genomes
ANXA2: Annexin A2

## References

[1] Bray F, Ferlay J, Soerjomataram I, Siegel RL, Torre LA, Jemal A. Global cancer statistics 2018: GLOBOCAN estimates of incidence and mortality worldwide for 36 cancers in 185 countries. CA Cancer J Clin. 2018;68:394–424. 30207593 10.3322/caac.21492

[2] Venkatesh HS, Morishita W, Geraghty AC, Silverbush D, Gillespie SM, Arzt M, Tam LT, Espenel C, Ponnuswami A, Ni L, et al. Electrical and synaptic integration of glioma into neural circuits. Nature. 2019;573:539–45. 31534222 10.1038/s41586-019-1563-y PMC7038898

[3] Louis DN, Perry A, Wesseling P, Brat DJ, Cree IA, Figarella-Branger D, Hawkins C, Ng HK, Pfister SM, Reifenberger G, et al. The 2021 WHO classification of tumors of the central nervous system: a summary. Neuro Oncol. 2021;23:1231–51. 34185076 10.1093/neuonc/noab106 PMC8328013

[4] Wesseling P, Capper D. WHO 2016 classification of gliomas. Neuropathol Appl Neurobiol. 2018;44:139–50. 28815663 10.1111/nan.12432

[5] Yang K, Wu Z, Zhang H, Zhang N, Wu W, Wang Z, Dai Z, Zhang X, Zhang L, Peng Y, et al. Glioma targeted therapy: insight into future of molecular approaches. Mol Cancer. 2022;21:39. 35135556 10.1186/s12943-022-01513-z PMC8822752

[6] Xue C, Li G, Zheng Q, Gu X, Bao Z, Lu J, Li L. The functional roles of the circRNA/Wnt axis in cancer. Mol Cancer. 2022;21:108. 35513849 10.1186/s12943-022-01582-0 PMC9074313

[7] Hu C, Xia R, Zhang X, Li T, Ye Y, Li G, He R, Li Z, Lin Q, Zheng S, Chen R. circFARP1 enables cancer-associated fibroblasts to promote gemcitabine resistance in pancreatic cancer via the LIF/STAT3 axis. Mol Cancer. 2022;21:24. 35045883 10.1186/s12943-022-01501-3 PMC8767726

[8] Pan Z, Zhao R, Li B, Qi Y, Qiu W, Guo Q, Zhang S, Zhao S, Xu H, Li M, et al. EWSR1-induced circNEIL3 promotes glioma progression and exosome-mediated macrophage immunosuppressive polarization via stabilizing IGF2BP3. Mol Cancer. 2022;21:16. 35031058 10.1186/s12943-021-01485-6 PMC8759291

[9] Vo JN, Cieslik M, Zhang Y, Shukla S, Xiao L, Zhang Y, Wu YM, Dhanasekaran SM, Engelke CG, Cao X, et al. The landscape of circular RNA in cancer. Cell. 2019;176:869–81. 30735636 10.1016/j.cell.2018.12.021 PMC6601354

[10] Xu S, Luo C, Chen D, Tang L, Cheng Q, Chen L, Liu Z. circMMD reduction following tumor treating fields inhibits glioblastoma progression through FUBP1/FIR/DVL1 and miR-15b-5p/FZD6 signaling. J Exp Clin Cancer Res. 2023;42:64. 36932454 10.1186/s13046-023-02642-z PMC10021944

[11] Cui Y, Wu X, Jin J, Man W, Li J, Li X, Li Y, Yao H, Zhong R, Chen S, et al. CircHERC1 promotes non-small cell lung cancer cell progression by sequestering FOXO1 in the cytoplasm and regulating the miR-142-3p-HMGB1 axis. Mol Cancer. 2023;22:179. 37932766 10.1186/s12943-023-01888-7 PMC10626661

[12] Jiang Y, Zhao J, Li R, Liu Y, Zhou L, Wang C, Lv C, Gao L, Cui D. CircLRFN5 inhibits the progression of glioblastoma via PRRX2/GCH1 mediated ferroptosis. J Exp Clin Cancer Res. 2022;41:307. 36266731 10.1186/s13046-022-02518-8 PMC9583503

[13] Wang Y, Wang B, Zhou F, Lv K, Xu X, Cao W. CircNDC80 promotes glioblastoma multiforme tumorigenesis via the miR-139-5p/ECE1 pathway. J Transl Med. 2023;21:22. 36635757 10.1186/s12967-022-03852-3 PMC9837923

[14] Wei Y, Lu C, Zhou P, Zhao L, Lyu X, Yin J, Shi Z, You Y. EIF4A3-induced circular RNA ASAP1 promotes tumorigenesis and temozolomide resistance of glioblastoma via NRAS/MEK1/ERK1-2 signaling. Neuro Oncol. 2021;23:611–24. 32926734 10.1093/neuonc/noaa214 PMC8041353

[15] Wang C, Zhang M, Liu Y, Cui D, Gao L, Jiang Y. CircRNF10 triggers a positive feedback loop to facilitate progression of glioblastoma via redeploying the ferroptosis defense in GSCs. J Exp Clin Cancer Res. 2023;42:242. 37723588 10.1186/s13046-023-02816-9 PMC10507871

[16] Zhou WY, Cai ZR, Liu J, Wang DS, Ju HQ, Xu RH. Circular RNA: metabolism, functions and interactions with proteins. Mol Cancer. 2020;19:172. 33317550 10.1186/s12943-020-01286-3 PMC7734744

[17] Wu P, Mo Y, Peng M, Tang T, Zhong Y, Deng X, Xiong F, Guo C, Wu X, Li Y, et al. Emerging role of tumor-related functional peptides encoded by lncRNA and circRNA. Mol Cancer. 2020;19:22. 32019587 10.1186/s12943-020-1147-3 PMC6998289

[18] Huang B, Ren J, Ma Q, Yang F, Pan X, Zhang Y, Liu Y, Wang C, Zhang D, Wei L, et al. A novel peptide PDHK1-241aa encoded by circPDHK1 promotes ccRCC progression via interacting with PPP1CA to inhibit AKT dephosphorylation and activate the AKT-mTOR signaling pathway. Mol Cancer. 2024;23:34. 38360682 10.1186/s12943-024-01940-0 PMC10870583

[19] Wu X, Xiao S, Zhang M, Yang L, Zhong J, Li B, Li F, Xia X, Li X, Zhou H, et al. A novel protein encoded by circular SMO RNA is essential for Hedgehog signaling activation and glioblastoma tumorigenicity. Genome Biol. 2021;22:33. 33446260 10.1186/s13059-020-02250-6 PMC7807754

[20] Song J, Zheng J, Liu X, Dong W, Yang C, Wang D, Ruan X, Zhao Y, Liu L, Wang P, et al. A novel protein encoded by ZCRB1-induced circHEATR5B suppresses aerobic glycolysis of GBM through phosphorylation of JMJD5. J Exp Clin Cancer Res. 2022;41:171. 35538499 10.1186/s13046-022-02374-6 PMC9086421

[21] Duan JL, Chen W, Xie JJ, Zhang ML, Nie RC, Liang H, Mei J, Han K, Xiang ZC, Wang FW, et al. A novel peptide encoded by N6-methyladenosine modified circMAP3K4 prevents apoptosis in hepatocellular carcinoma. Mol Cancer. 2022;21:93. 35366894 10.1186/s12943-022-01537-5 PMC8976336

[22] Meng X, Zhao Y, Han B, Zha C, Zhang Y, Li Z, Wu P, Qi T, Jiang C, Liu Y, Cai J. Dual functionalized brain-targeting nanoinhibitors restrain temozolomide-resistant glioma via attenuating EGFR and MET signaling pathways. Nat Commun. 2020;11:594. 32001707 10.1038/s41467-019-14036-x PMC6992617

[23] Li Z, Huang C, Bao C, Chen L, Lin M, Wang X, Zhong G, Yu B, Hu W, Dai L, et al. Exon-intron circular RNAs regulate transcription in the nucleus. Nat Struct Mol Biol. 2015;22:256–64. 25664725 10.1038/nsmb.2959

[24] Chen Y, Ling Z, Cai X, Xu Y, Lv Z, Man D, Ge J, Yu C, Zhang D, Zhang Y, et al. Activation of YAP1 by N6-methyladenosine-modified circCPSF6 drives malignancy in hepatocellular carcinoma. Cancer Res. 2022;82:599–614. 34916222 10.1158/0008-5472.CAN-21-1628

[25] Wu A, Hu Y, Xu Y, Xu J, Wang X, Cai A, Liu R, Chen L, Wang F. Methyltransferase-like 3-mediated m6A methylation of Hsa_circ_0058493 accelerates hepatocellular carcinoma progression by binding to YTH domain-containing protein 1. Front Cell Dev Biol. 2021;9: 762588. 34888309 10.3389/fcell.2021.762588 PMC8650312

[26] Ji F, Lu Y, Chen S, Yu Y, Lin X, Zhu Y, Luo X. IGF2BP2-modified circular RNA circARHGAP12 promotes cervical cancer progression by interacting m(6)A/FOXM1 manner. Cell Death Discov. 2021;7:215. 34392306 10.1038/s41420-021-00595-w PMC8364552

[27] Huang Q, Guo H, Wang S, Ma Y, Chen H, Li H, Li J, Li X, Yang F, Qiu M, et al. A novel circular RNA, circXPO1, promotes lung adenocarcinoma progression by interacting with IGF2BP1. Cell Death Dis. 2020;11:1031. 33268793 10.1038/s41419-020-03237-8 PMC7710735

[28] Chen X, Zhu S, Li HD, Wang JN, Sun LJ, Xu JJ, Hui YR, Li XF, Li LY, Zhao YX, et al. N(6)-methyladenosine-modified circIRF2, identified by YTHDF2, suppresses liver fibrosis via facilitating FOXO3 nuclear translocation. Int J Biol Macromol. 2023;248: 125811. 37467831 10.1016/j.ijbiomac.2023.125811

[29] Wang D, Guan H, Xia Y. YTHDC1 maintains trophoblasts function by promoting degradation of m6A-modified circMPP1. Biochem Pharmacol. 2023;210: 115456. 36780989 10.1016/j.bcp.2023.115456

[30] Chen RX, Chen X, Xia LP, Zhang JX, Pan ZZ, Ma XD, Han K, Chen JW, Judde JG, Deas O, et al. N(6)-methyladenosine modification of circNSUN2 facilitates cytoplasmic export and stabilizes HMGA2 to promote colorectal liver metastasis. Nat Commun. 2019;10:4695. 31619685 10.1038/s41467-019-12651-2 PMC6795808

[31] Lin Z, Lv D, Liao X, Peng R, Liu H, Wu T, Wu K, Sun Y, Zhang Z. CircUBXN7 promotes macrophage infiltration and renal fibrosis associated with the IGF2BP2-dependent SP1 mRNA stability in diabetic kidney disease. Front Immunol. 2023;14:1226962. 37744330 10.3389/fimmu.2023.1226962 PMC10516575

[32] Wang L, Zhou J, Zhang C, Chen R, Sun Q, Yang P, Peng C, Tan Y, Jin C, Wang T, et al. A novel tumour suppressor protein encoded by circMAPK14 inhibits progression and metastasis of colorectal cancer by competitively binding to MKK6. Clin Transl Med. 2021;11: e613. 34709743 10.1002/ctm2.613 PMC8516360

[33] Liu H, Fang D, Zhang C, Zhao Z, Liu Y, Zhao S, Zhang N, Xu J. Circular MTHFD2L RNA-encoded CM-248aa inhibits gastric cancer progression by targeting the SET-PP2A interaction. Mol Ther. 2023;31:1739–55. 37101395 10.1016/j.ymthe.2023.04.013 PMC10277894

[34] Koh M, Lim H, Jin H, Kim M, Hong Y, Hwang YK, Woo Y, Kim ES, Kim SY, Kim KM, et al. ANXA2 (annexin A2) is crucial to ATG7-mediated autophagy, leading to tumor aggressiveness in triple-negative breast cancer cells. Autophagy. 2024;20:659–74. 38290972 10.1080/15548627.2024.2305063 PMC10936647

[35] Buttarelli M, Babini G, Raspaglio G, Filippetti F, Battaglia A, Ciucci A, Ferrandina G, Petrillo M, Marino C, Mancuso M, et al. A combined ANXA2-NDRG1-STAT1 gene signature predicts response to chemoradiotherapy in cervical cancer. J Exp Clin Cancer Res. 2019;38:279. 31242951 10.1186/s13046-019-1268-y PMC6595690

[36] Wang Y, Wang Y, Liu W, Ding L, Zhang X, Wang B, Tong Z, Yue X, Li C, Xu L, et al. TIM-4 orchestrates mitochondrial homeostasis to promote lung cancer progression via ANXA2/PI3K/AKT/OPA1 axis. Cell Death Dis. 2023;14:141. 36806050 10.1038/s41419-023-05678-3 PMC9941510

[37] Chaudhary P, Thamake SI, Shetty P, Vishwanatha JK. Inhibition of triple-negative and Herceptin-resistant breast cancer cell proliferation and migration by Annexin A2 antibodies. Br J Cancer. 2014;111:2328–41. 25321192 10.1038/bjc.2014.542 PMC4264449

[38] Boerner JL, Demory ML, Silva C, Parsons SJ. Phosphorylation of Y845 on the epidermal growth factor receptor mediates binding to the mitochondrial protein cytochrome c oxidase subunit II. Mol Cell Biol. 2004;24:7059–71. 15282306 10.1128/MCB.24.16.7059-7071.2004 PMC479738

[39] Song H, Huang L, Zhang M, Wang X, Song S, Yang L. Transphosphorylation of EGFR at Y845 plays an important role in its autophosphorylation and kinase activity. Oncol Rep. 2014;31:2393–8. 24677053 10.3892/or.2014.3102

[40] Sato K. Cellular functions regulated by phosphorylation of EGFR on Tyr845. Int J Mol Sci. 2013;14:10761–90. 23702846 10.3390/ijms140610761 PMC3709701

[41] Pidugu VK, Wu MM, Yen AH, Pidugu HB, Chang KW, Liu CJ, Lee TC. IFIT1 and IFIT3 promote oral squamous cell carcinoma metastasis and contribute to the anti-tumor effect of gefitinib via enhancing p-EGFR recycling. Oncogene. 2019;38:3232–47. 30626937 10.1038/s41388-018-0662-9

[42] Chen W, Xu J, Wu Y, Liang B, Yan M, Sun C, Wang D, Hu X, Liu L, Hu W, et al. The potential role and mechanism of circRNA/miRNA axis in cholesterol synthesis. Int J Biol Sci. 2023;19:2879–96. 37324939 10.7150/ijbs.84994 PMC10266072

[43] Xiong L, Liu HS, Zhou C, Yang X, Huang L, Jie HQ, Zeng ZW, Zheng XB, Li WX, Liu ZZ, et al. A novel protein encoded by circINSIG1 reprograms cholesterol metabolism by promoting the ubiquitin-dependent degradation of INSIG1 in colorectal cancer. Mol Cancer. 2023;22:72. 37087475 10.1186/s12943-023-01773-3 PMC10122405

[44] Peng Y, Xu Y, Zhang X, Deng S, Yuan Y, Luo X, Hossain MT, Zhu X, Du K, Hu F, et al. A novel protein AXIN1-295aa encoded by circAXIN1 activates the Wnt/beta-catenin signaling pathway to promote gastric cancer progression. Mol Cancer. 2021;20:158. 34863211 10.1186/s12943-021-01457-w PMC8642992

[45] Ma S, Chen C, Ji X, Liu J, Zhou Q, Wang G, Yuan W, Kan Q, Sun Z. The interplay between m6A RNA methylation and noncoding RNA in cancer. J Hematol Oncol. 2019;12:121. 31757221 10.1186/s13045-019-0805-7 PMC6874823

[46] Lin H, Wang Y, Wang P, Long F, Wang T. Mutual regulation between N6-methyladenosine (m6A) modification and circular RNAs in cancer: impacts on therapeutic resistance. Mol Cancer. 2022;21:148. 35843942 10.1186/s12943-022-01620-x PMC9290271

[47] Shao Y, Liu Z, Song X, Sun R, Zhou Y, Zhang D, Sun H, Huang J, Wu C, Gu W, et al. ALKBH5/YTHDF2-mediated m6A modification of circAFF2 enhances radiosensitivity of colorectal cancer by inhibiting Cullin neddylation. Clin Transl Med. 2023;13: e1318. 37381158 10.1002/ctm2.1318 PMC10307995

[48] Guo G, Gong K, Wohlfeld B, Hatanpaa KJ, Zhao D, Habib AA. Ligand-independent EGFR signaling. Cancer Res. 2015;75:3436–41. 26282175 10.1158/0008-5472.CAN-15-0989 PMC4558210

[49] Lemmon MA, Schlessinger J. Cell signaling by receptor tyrosine kinases. Cell. 2010;141:1117–34. 20602996 10.1016/j.cell.2010.06.011 PMC2914105

[50] Shostak K, Chariot A. EGFR and NF-kappaB: partners in cancer. Trend Mol Med. 2015;21:385–93. 10.1016/j.molmed.2015.04.001 25979753

[51] Sigismund S, Avanzato D, Lanzetti L. Emerging functions of the EGFR in cancer. Mol Oncol. 2018;12:3–20. 29124875 10.1002/1878-0261.12155 PMC5748484

[52] Bahl S, Ling H, Acharige N, Santos-Barriopedro I, Pflum M, Seto E. EGFR phosphorylates HDAC1 to regulate its expression and anti-apoptotic function. Cell Death Dis. 2021;12:469. 33976119 10.1038/s41419-021-03697-6 PMC8113371

