# Supplementary material for: A novel protein SPECC1-415aa encoded by N6-methyladenosine modified circSPECC1 regulates the sensitivity of glioblastoma to TMZ
Source: Cell Mol Biol Lett. 2024 Sep 27;29:127. doi: 10.1186/s11658-024-00644-z (PMC11429730; doi:10.1186/s11658-024-00644-z)
Supplement: Supplementary file 1 — Supplementary Material 1. [file 11658_2024_644_MOESM1_ESM.docx]

**A novel protein SPECC1-415aa encoded by N6-methyladenosine modified circSPECC1 regulates the sensitivity of glioblastoma to TMZ**

**Cheng Wei^1, #^, Dazhao Peng^1, #^, Boyuan Jing^1, #^, Bo Wang^1^, Zesheng Li^1^, Runze Yu^1^, Su Zhang^1^, Jinquan Cai^3, *^, Zhenyu Zhang^2, *^, Jianning Zhang ^1, *^, Lei Han^1, *^**

1 Tianjin Neurological Institute, Key Laboratory of Post-Neuroinjury Neuro-repair and Regeneration in Central Nervous System, Ministry of Education and Tianjin City, Tianjin Medical University General Hospital, Tianjin, 300052, China.

2 Department of Neurosurgery, The First Affiliated Hospital of Zhengzhou University, Zhengzhou, Henan, China.

3 Department of Neurosurgery, The Second Affiliated Hospital of Harbin Medical University, Harbin, China.

**Supplementary Materials**

**Materials and Methods:**

1. **CircRNAs microarray hybridization and transcriptome analyses**

The circRNAs microarray of four primary human brain GBM tissue samples and four recurrent human brain GBM tissue samples were performed at Aksomics Biotechnology Co., LTD (Shanghai, China). The total RNA was qualified and subsequently digested with RNase R (Epicentre, Inc.) to remove linear RNAs and enrich circRNAs. Then, the enriched circRNAs were amplified and transcribed into fluorescent complementary RNA (cRNA) utilizing a random priming method (Arraystar Super RNA Labeling Kit; Arraystar). The labeled cRNAs were hybridized onto the Arraystar Human circRNA Array V2 (8x15K, Arraystar). After having washed the slides, the arrays were scanned by the Agilent Scanner G2505C. Agilent Feature Extraction software (version 11.0.1.1) was used to analyze acquired array images. Quantile normalization and subsequent data processing were performed using the R software limma package. By circRNAs microarray, a total of 7367 circRNAs that were upregulated in recurrent GBM and 6250 circRNAs that were downregulated in recurrent GBM were obtained. According to the filter condition |FC|≥1.3 and *P*<0.05, the circRNAs microarray identified 412 significantly upregulated circRNAs and 173 significantly downregulated circRNAs in recurrent GBM compared with primary GBM.

The transcriptome in this study were performed at Gene DenovoBiotechnology Co., LTD (Guangzhou, China). Total RNA was extracted using Trizol reagent kit (Invitrogen, Carlsbad, CA, USA) according to the manufacturer’s protocol. RNA quality was assessed on an Agilent 2100 Bioanalyzer (Agilent Technologies, Palo Alto, CA, USA) and checked using RNase free agarose gel electrophoresis. After total RNA was extracted, eukaryotic mRNA was enriched by Oligo(dT) beads. Then the enriched mRNA was fragmented into short fragments using fragmentation buffer and reversly transcribed into cDNA by using NEBNext Ultra RNA Library Prep Kit for Illumina (NEB #7530, New England Biolabs, Ipswich, MA, USA). The ligation reaction was purified with the AMPure XP Beads (1.0X). And polymerase chain reaction (PCR) amplified. The resulting cDNA library was sequenced using Illumina Novaseq6000. Transcriptome sequencing was performed in LN229 GBM cell line after knocking down circSPECC1 after 48 hours. A total of 196 significantly upregulated mRNAs and 335 significantly downregulated mRNAs were identified in LN229 GBM cells with circSPECC1 knockdown compared with control group (|FC|≥2, *P*＜0.05 and FDR＜0.05). Next, in order to investigate the major targets and signaling pathways regulated by circSPECC1, GO functional annotation and KEGG pathway enrichment analysis were carried out on the differentially expressed mRNAs acquired by transcriptome via SangerBox portal (http://vip.sangerbox.com/home.html).

1. **Analysis of bioinformatics and databases**

Through circBank (http://www.circbank.cn/index.htmL), circRNADb (http://repr O.org/transcirc/od.njmu.edu.cn/cgi-bin/circrnadb/circRNADb.php) and TransCirc (https://www.biosin) databases, the coding potential of Top10 upregulated circRNAs in recurrent GBM samples and Top10 downregulated circRNAs in recurrent GBM samples were analyzed. The screening criteria were as follows: circBank (coding_prob>0.3); circRNADb (R Score>1.5); TransCirc (Evidence score>2). The Top 11 differentially expressed circRNAs with coding potential were obtained for further study. By RNAfold ([http://rna.tbi.univie.ac.at/cgi-bin/RNAWebSuite/R NAfold.Cgi](http://rna.tbi.univie.ac.at/cgi-bin/RNAWebSuite/R%20NAfold.Cgi)) prediction software, the internal ribosomal entry sites (IRES) secondary structures of hsa_circ_0000745, hsa_circ_0002538 and hsa_circ_0007940 were analyzed. The complementarity of 18S ribosomal RNA (18S rRNA) on IRES and the characteristics of the 40-60 nt SuRE element on IRES were analyzed. The raw data of circRNA sequencing datasets GSE86202, GSE109569, GSE92322, and GSE165926 were obtained from the GEO database (https://www.ncbi.nl m.nih.gov/geo/). The GSE86202 and GSE109569 datasets were subjected to sequencing analysis using three non-tumor brain tissue samples (NTT) and three glioblastoma samples (GBM). For the GSE92322 dataset, five NTT samples and five GBM samples were used for sequencing analysis. For the GSE165926 dataset, 4 NTT samples and 11 high-grade glioma (HGG) samples were used for sequencing analysis.

Through SRAMP online website (http://www.cuilab.cn/sramp), we forecasted the m^6^A modification sites on circSPECC1. Combined Score can be obtained in the prediction process, which can be divided into four levels of low confidence, moderate confidence, high confidence and very high confidence. Furthermore, four very high confidence m^6^A modification sites on circSPECC1 were screened according to Combined Score > 0.8.

1. **SiRNA, plasmid transfection and lentivirus infection**

SiRNAs for circSPECC1 knockdown were purchased from Shanghai Hanbio Biotechnology Co., LTD. (China). The circ_0000745, circ_0002538, circ_0007940 overexpression plasmids with 3×Flag tag and circSPECC1 (circ_0000745) overexpression plasmids were constructed from Genewiz Biotechnology Co., LTD (Suzhou, China). Lv-circSPECC1, Lv-control and luciferase lentiviruses were purchased from Genechem Technology Co., LTD (Shanghai, China). Lv-circSPECC1 was used for stable overexpression of circSPECC1. Lipofectamine 3000 (L3000001) reagent for plasmid transfection and Lipofectamine RNAiMAX (13778030) reagent for siRNA transfection were purchased from Thermo Fisher Scientific company (USA). The conditions of siRNAs and plasmids transfection and virus infection were referred to the articles already published by our research group[1]. The Supplementary Table 1 displayed the siRNA sequences.

1. **Total RNA extraction, reverse transcription (RT) and quantitative real-time PCR (qPCR)**

TRIzol reagent (9109, Takara, Japan) was used to extract total RNA. Using a GoScript Reverse Transcription system (A5001, Promega, USA), the mRNAs of IGF2BP1/2/3 and circRNA of circ_0000745 and circ_0007940 were reverse transcribed in order to synthesize cDNA. The GoTaq® qPCR Master Mix (A6001, Promega, USA) test kit was utilized to measure the expression status of mRNA and circRNA on an ABI QuantStudio 3, with GAPDH expression serving as an internal control. The PCR system used for mRNA and circRNA was 20 μL, and the following steps were carried out: 40 cycles of 95 °C for 15 s and 60 °C for 1 min, followed by 10 min at 95 °C. The program for the dissolution curve was: 95 °C for 15 s, 60 °C for 1 min, and 95 °C for 1 s. By using 2^−△△CT^, the relative expression levels of the mRNA and circRNA were determined. Supplementary Table 1 showed the primer sequences.

1. **Western blot assay**

RIPA lysis buffer (Solarbio, China) containing protease and phosphatase inhibitor (Sigma-Aldrich, USA) was used to lyse the cells. Following the manufacturer's instructions, a BCA kit (Solarbio, China) was used to determine the protein concentration. The 10% SDS-PAGE gels were used to separate the proteins and the 0.22 μm PVDF membrane (Millipore, USA) were used to transfer the proteins. The membranes were incubated with particular antibodies overnight at 4 °C after being blocked with BSA for two hours at room temperature. The antibodies used were: Flag (#14793, Cell Signaling), GAPDH (#2118, Cell Signaling), SPECC1-415aa (13851-1-AP, Proteintech), IGF2BP1 (sc-166344, Santa Cruz), ANXA2 (sc-28385, Santa Cruz), EGFR (#4267, Cell Signaling), AKT (#9272, Cell Signaling), p-EGFR (Tyr845) (#2231, Cell Signaling) and p-AKT (Ser473) (#4060, Cell Signaling). After that, the membranes were incubated with the matching secondary antibody that was HRP-conjugated. Then, the enhanced chemiluminescence system (Bio-Rad, USA) was used to identify the protein bands. The gray value of the protein bands was calculated using Image J software, and the relative gray value was obtained by using the gray value of the target protein /GAPDH. Western blot assay was conducted as previously described[1].

1. **Dual luciferase assay**

The dual luciferase reporter gene was constructed by chemical gene synthesis (Genewiz, China). Through the psicheck2 vector, the IRES sequences of circSPECC1, as well as the truncated IRES sequences of circSPECC1, were amplified and inserted into the middle of renal luciferase (Rluc) and luciferase (Luc) through two restriction enzyme sites, NotI and MluI. The IRES sequence was deleted from the negative control plasmid. IRES activities were verified using a dual luciferase reporter system (E1910, Promega). The luciferase activity of Luc relative to RLuc (Luc/RLuc) in each sample was detected.

1. **Actinomycin D assay**

Actinomycin D (Act D, HY-17559) reagent used in the study was purchased from MedChemExpress (MCE) company. First, the cells were seeded into 6-well plates (2×10^5^ cells per well). Act D reagent (6.28 µg/µL) was added to 2 mL medium per well of 6-well plates at 1.5µg/mL medium. Cells were collected at different time points: 0 h, 2 h, 4 h, 8 h and 12 h, and then RNA extraction and RT-qPCR experiments were performed to detect RNA expression levels.

1. **RNase R assay**

RNase R used in this study was purchased from Abcam company (UK). The total RNA and 2.5 U/μg RNase R were incubated at 37 °C for 20 min. After the RNase R treatment, the RNA expression levels of circSPECC1 were analysed by RT-qPCR.

1. **Isolation of nuclear and cytoplasmic RNA**

To prevent RNA degradation and inhibit RNA enzyme activity, RNA enzyme inhibitor (N2615, Promega, USA) was utilized. The 0.5% NP-40 and 0.1% NP-40 (85124, Thermo Fisher Scientific) were used to isolate nuclear and cytoplasmic RNA. Subsequent RT-qPCR experiments were conducted using nuclear and cytoplasmic extracts, as previously mentioned.

1. **RNA fluorescence in situ hybridization (RNA FISH)**

The GenePharma Co., Ltd. provided the Cy3-labeled circSPECC1 probes (Shanghai, China). The RNA FISH Kit (GenePharma, China) was used for hybridizations in accordance with the manufacturer's instructions. The glioblastoma cells were fixed with 4% paraformaldehyde and then treated with 0.5% Triton. After that, cells were grown using particular probes for the overnight. The IX81 fluorescence inverted microscope (Olympus, Japan) was used to take all of the fluorescence images.

1. **IC50 assay**

The experiments were conducted three times for each sample using 96 wells (6×10^3^ cell/well) seeded with LN229, U251, LN229R, and U251R cells. For 48 hours, the cells were incubated with temozolomide (TMZ) at varying concentrations. Subsequently, cell viability was evaluated using the Cell Counting Kit-8 (CCK-8) Kit (APE×BIO, USA).

1. **CCK-8 assay**

CCK-8 reagent (K1018) was purchased from APE×BIO company, USA. LN229, U251, LN229R and U251R cells were seeded in 96 wells. After 24h of incubation in a cell thermostat incubator at 37 °C, siRNAs for circSPECC1 knockdown or plasmids for circSPECC1 overexpression were added to the wells. 24 hours later, the test was started, and the test was performed for 5 consecutive days (0, 1, 2, 3, 4, 5 days). Using a multimode microplate reader (Synergy2, BioTek, USA), the optical density (OD) at 450 nm wavelength was determined.

1. **Wound healing and Transwell experiments**

The GBM cells were seeded at a density of 2×10^5^ cells per well in 6-well plates for the wound healing experiment. Transfection of plasmids or siRNAs was done after 24 h. After 24 h, a 200 μL pipetting head was used to scuff the cells in the plate. Using an inverted microscope (IX81, Olympus Company, Japan), images were taken at 0 h, 12 h, and 24 h. ImageJ software was then used to calculate the cell migration rate. Matrigel matrix glue (Corning, USA) was applied to the upper chamber of the Transwell experiment. Once the upper chamber had solidified for 40 min at 37 °C in a cell culture incubator, it was transferred to a 24-well plate. Subsequently, 200 μL of serum-free medium was used to seed 5×10^4^ cells into the upper chamber, and 500 μL of full medium containing 10% fetal bovine serum (FBS) was added to the lower chamber. Following a 24 h culture period, cells were fixed for 10 min with formaldehyde and stained for 10 s with 1% crystal violet. A positive microscope (BX53, Olympus Company, Japan) was used to collect images, and the quantity of gelled cells was counted.

1. **Colony formation assay**

The 5×10^2^ GBM cells with circSPECC1 knockdown or overexpression were seeded into 6-well plates in 2 mL medium and the cells were observed every 3 days. The colony formation was observed at 10-15 days, and the clone point with more than 50 cells could be counted as a colony, and the experiment was terminated at 10-15 days.

After being fixed for 30 min with 1 mL of 4% paraformaldehyde, the cells were stained for 10 min with 600 µL of 2.5% crystal violet. The 6-well plate was panned 3 times in clear water, and images were collected after drying.

1. **Immunofluorescence assay**

GBM cells were cultivated with 500 µL of full medium after being seeded at a density of 2×10^4^ cells per well in a 24-well plate. After 24 h, the cell morphology was observed and the degree of cell confluence was observed. Then, siRNAs or plasmids were used to downregulated or upregulated the expression of circSPECC1. After being fixed for 15 min with 4% paraformaldehyde, the cells were incubated for 20 min at room temperature with 0.5% TritonX-100. Furthermore, cells were blocked for 30 min using 5% BSA. 150 µL of primary γH2AX antibody (ab26350, abcam) diluted with antibody diluent was added to each well and cells were incubated overnight at 4 °C in the dark. Fluorescent secondary antibody (sheep anti-mouse secondary antibody) diluted in antibody diluent was added, and cells were incubated at 37 ℃ for 1 h in the dark. After the incubation of the secondary antibody, the cover slip was placed onto a slide containing DAPI mounting medium (ab104139, abcam). Finally, using an IX3 fluorescence inverted microscope (Olympus, Japan), pictures were taken.

1. **Comet assay**

Following the manufacturer's instructions, the Comet Assay DNA damage detection kit (KGA240-50, KeyGEN, China) was used to conduct the assay. Using an IX3 fluorescence inverted microscope (Olympus, Japan), pictures were taken.

1. **EdU experiment**

Ribobio Co., Ltd. (Guangzhou, China) was the source of the EdU assay Kit (Cell-Light EDU Apollo567 In Vitro Kit). Cells were seeded into 96-well plates (corresponding to 200 μL of medium) at 2×10^4^ cells per well, and subsequent experiments were performed after 24 h to 48 h. After diluting the EdU solution with full medium at a 1000:1 ratio, 100 μL of 50 μM EdU culture medium was added to each well, and the wells were incubated for 2 h at 37 °C. 50 μL of cell fixative solution (4% paraformaldehyde) was used to fix the cells, which were then incubated for 30 min at room temperature. Following the addition of 100 μL of 0.5% TritonX-100, the wells were incubated for 10 min. The wells were filled with 100 μL of 1×Appolo staining reaction solution, and they were left in the dark for 30 min.

Then, each well received 100 μL of the 1×Hoechst reaction solution, which was then left to sit at room temperature in the dark for 10 min. The EdU positive rates were computed using fluorescence images obtained with an IX3 inverted fluorescence microscope (Olympus, Japan).

1. **Immunoprecipitation and mass spectrometry (IP-MS)**

The Flag antibody (66008-4-lg) used during the IP experiment was purchased from Proteintech company. SPECC1-415aa antibody (13851-1-AP, Proteintech) was used to endogenous IP experiment. We bought Protein A/G agarose beads (sc-2003) from Santa Cruz Biotechnology. Protease inhibitors (78425, Thermo Fisher) were added to the IP lysate (R0100, Solarbio) at a ratio of 1:100, and 6×10^6^ cells were lysed using 1ml of IP lysate. The protein lysate concentration was identified through the BCA method, and the total protein in the incubation system was quantified as 2 mg/mL based on the protein lysate concentration measured. Then, 3 µg of target antibody and 60 µL protein A/G agarose beads were added to the above incubation system and incubated overnight at 4 °C in a turning shaker. 300 μL of prepared protein lysate was added to each tube and washed three times at 4 °C for 30 min each time. To each tube, 25 μL of IP lysate and 25 μL of 2×protein loading buffer (containing protease inhibitors) were added and boiled for 10 min at 98 °C. Finally, 10 μL of the obtained IP samples were removed from each tube and verified by Western blot assay. The remaining IP samples were subjected to mass spectrometry (MS) analysis following Western blot analysis.

1. **M^6^A RNA immunoprecipitation (MeRIP) and RNA immunoprecipitation (RIP) experiments**

The manufacturer's instructions for the Magna RIP^TM^ RNA-Binding Protein Immunoprecipitation Kit (17-700, Merck Millipore, USA) were followed. 4×10^7^ GBM cells were taken from the m^6^A and IGF2BP1 RIP experiments, and they were lysed in RIP lysis buffer. For anti-IP (anti-m^6^A or anti-IGF2BP1), anti-IgG (negative control), and input (positive control), each lysate was further split into three groups. To enrich RNA binding protein (RBP), m^6^A, IGF2BP1, or an IgG antibody were added to each sample. After that, dynabeads were used to gather the bound RNA and the relevant RBP. Following the removal of unbound material, Proteinase K was used to digest the RBP. The RNA that was immunoprecipitated and bound to the RBP was then purified and reverse-transcribed (RT) into cDNA. Afterwards, the percentage input of circSPECC1 in each group was determined using a qPCR assay. Supplementary Tables 1 contained the primer sequences needed for the RIP analysis.

1. **TUNEL assay**

Beyotime Biotechnology Co., LTD (Shanghai, China) supplied the TUNEL cell apoptosis detection kit (C1088). Initially, 2×10^4^ cells were seeded into each well of 24-well plates. Each well was fixed using 500 μL of 4% tissue fixative for 30 min. Then, 500 μL of 0.3% TritonX-100 was added to each well and incubated at room temperature for 5 min. The TUNLE reaction solution was prepared at a ratio of 5 μl:45 μl. Each well received 50 μL of the TUNEL reaction solution, which was then incubated for 60 min at 37 °C in the dark. Cell slides were mounted upside down onto slides dripped with DAPI mounting agent (ab104139, abcam). Finally, using an IX3 fluorescence inverted microscope (Olympus, Japan), pictures were taken.

1. **Intracranial tumor model construction**

The Animal Care and Use Committee of Tianjin Medical University approved the protocols, and all animal procedures were carried out in compliance with their guidelines. We bought female BALB/c nude mice at four weeks old from Hanfeng Biotechnology Co., LTD (Beijing, China). Luciferase lentivirus and Lv-circSPECC1 or Lv-control lentivirus were infected into LN229R cells in advance. The intracranial striatum of nude mice was injected with 5×10^5^ LN229R-luc-control or LN229R-luc-circSPECC1 cells using a stereotaxic apparatus and a microinfusion pump (Stoelting, USA). Both the tumor-bearing mice transplanted with LN229R-luc-control and the tumor-bearing mice transplanted with LN229R-luc-circSPECC1 were randomly split into two groups that consisted of ten mice each. The experiment was divided into 4 groups (control group, TMZ group, circSPECC1 group and circSPECC1 combined with TMZ group) with 10 mice in each group. On the 7th day after tumor cell implantation, TMZ treatment (5 mg/kg, intraperitoneal injection) was given in the TMZ group and the circSPECC1 combined with TMZ group. After continuous use for 5 days, the drug was stopped for 2 days and a total of 2 cycles of treatment were performed. Fluorescence imaging was performed every 7 days by an IVIS imaging system (perkinelmer, USA) to collect fluorescence intensity and monitor tumor growth. The survival mice were sacrificed on the 21st day. Kaplan-Meier survival curve was used for survival analysis. The brain tissues of nude mice in each group were collected for paraffin-embedded sections, and analyzed by HE staining and IHC staining.

1. **Hematoxylin and eosin (HE) staining and immunohistochemistry (IHC) staining**

Tumor tissues were preserved in 10% neutral buffered formalin for HE staining and IHC analysis prior to histological examination. HE staining was carried out using the HE staining kit (G1120, Solarbio) in accordance with the manufacturer's instructions, and an Olympus BX53 microscope was used to take the pictures. Graded alcohols were used to rehydrate 8 µm slides to distilled water (dH_2_O) before they were dewaxed in xylene for IHC analysis. Using sodium citrate (pH=6) buffer, antigen retrieval was carried out for 20 min at 97 °C. Slides were blocked for 30 min at room temperature using 5% blocking serum after being cleaned in PBS. After that, the slides were incubated with primary antibodies against Ki-67 (TA800648, ZSGB-BIO), p-EGFR (Tyr845) (#2231, Cell Signaling), SPECC1-415aa (13851-1-AP, Proteintech) and p-AKT (Ser473) (#4060, Cell Signaling) for an overnight period at 4 °C. Subsequently, the slides were incubated for one hour at 37 °C with a biotin-labeled secondary antibody, and they were incubated with diaminobenzidine (DAB). The slides were mounted and counterstained with hematoxylin and an Olympus BX53 microscope was used to take pictures.

**References:**

1. Wei C, Zhang X, Peng D, Zhang X, Guo H, Lu Y, Luo L, Wang B, Li Z, He Y, et al: **LncRNA HOXA11-AS promotes glioma malignant phenotypes and reduces its sensitivity to ROS via Tpl2-MEK1/2-ERK1/2 pathway.** *Cell Death Dis* 2022, **13:**942.


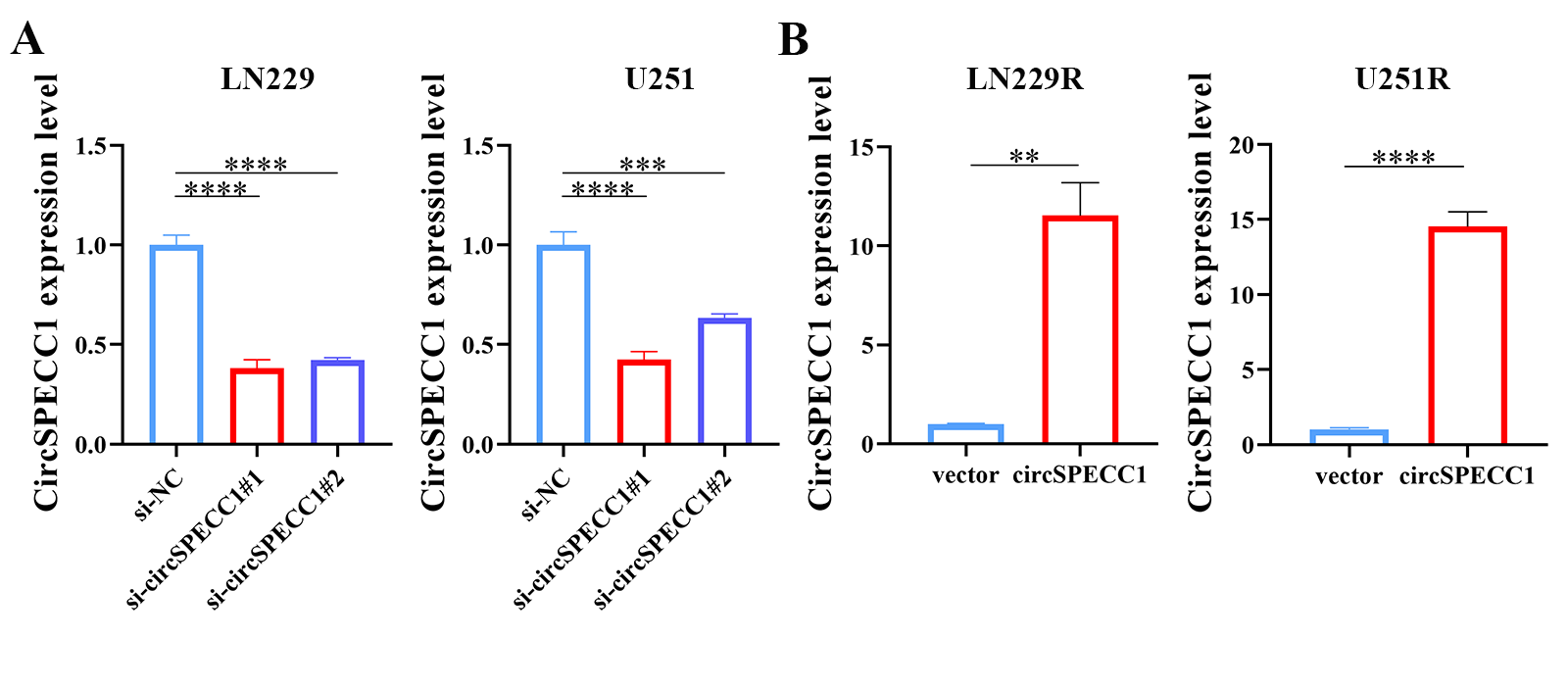


**Supplementary Figure 1. CircSPECC1 expression level in GBM and TMZ-resistant GBM cell lines after circSPECC1 knockdown or overexpression.** (A) RT-qPCR detection for circSPECC1 expression in LN229 and U251 cells after circSPECC1 knockdown. (B) RT-qPCR detection for circSPECC1 expression in LN229R and U251R cells after circSPECC1 overexpression.


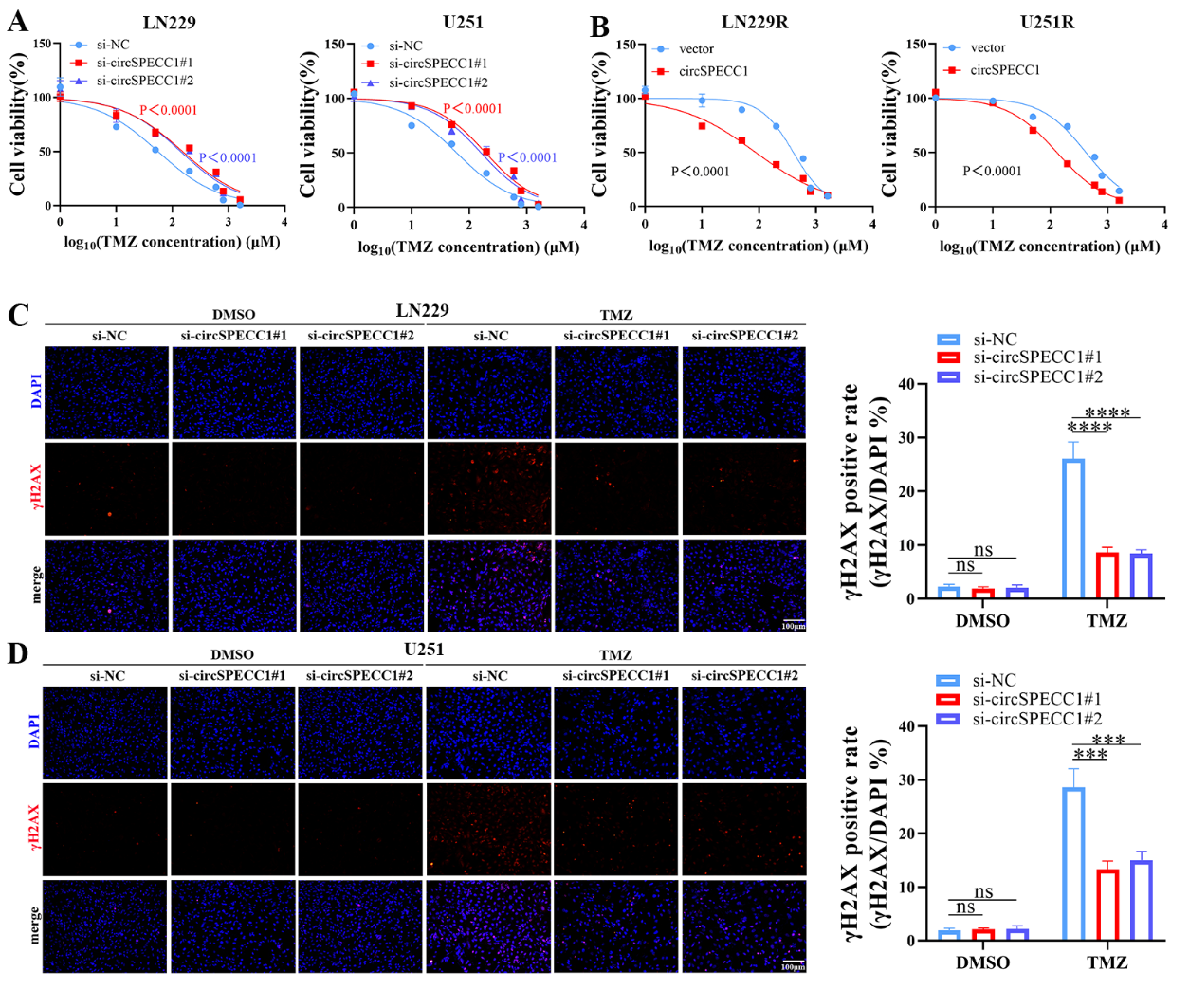


**Supplementary Figure 2. IC50 and γH2AX immunofluorescence assays with circSPECC1 knockdown or overexpression.** (A) Cell viability assay of circSPECC1 knockdown in LN229 and U251 cells treated with various concentrations of TMZ for 48 h. (B) Cell viability assay of circSPECC1 overexpression in LN229R and U251R cells treated with various concentrations of TMZ for 48 h. (C) Immunofluorescence assay for γH2AX expression in LN229 cells with circSPECC1 knockdown after DMSO or TMZ co-treatment. (D) Immunofluorescence assay for γH2AX expression in U251 cells with circSPECC1 knockdown after DMSO or TMZ co-treatment. ns, *P*＞0.05; ***, *P*＜0.001; ****, *P*＜0.0001.


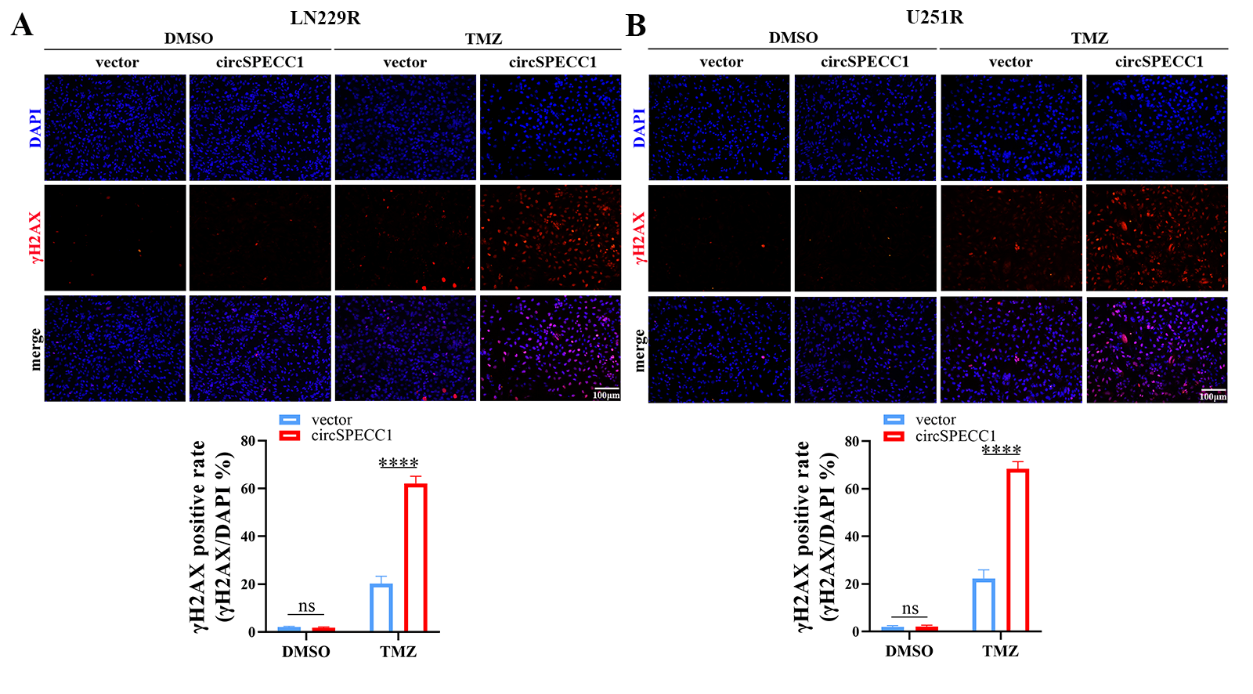


**Supplementary Figure 3. Immunofluorescence assays for γH2AX expression in LN229R and U251R cells with circSPECC1 overexpression after DMSO or TMZ co-treatment.** ns, *P*＞0.05; ****, *P*＜0.0001.


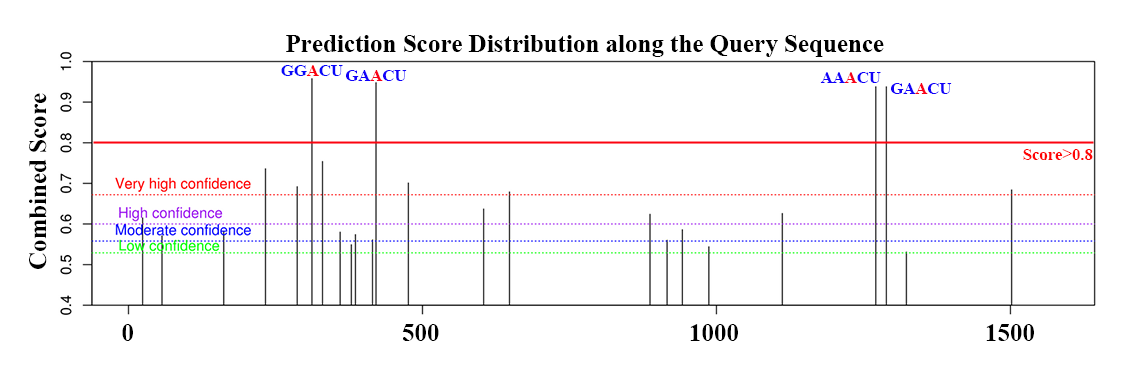


**Supplementary Figure 4. Prediction of m^6^A modification sites on circSPECC1 by SRAMP database (very high confidence: Score > 0.8)**


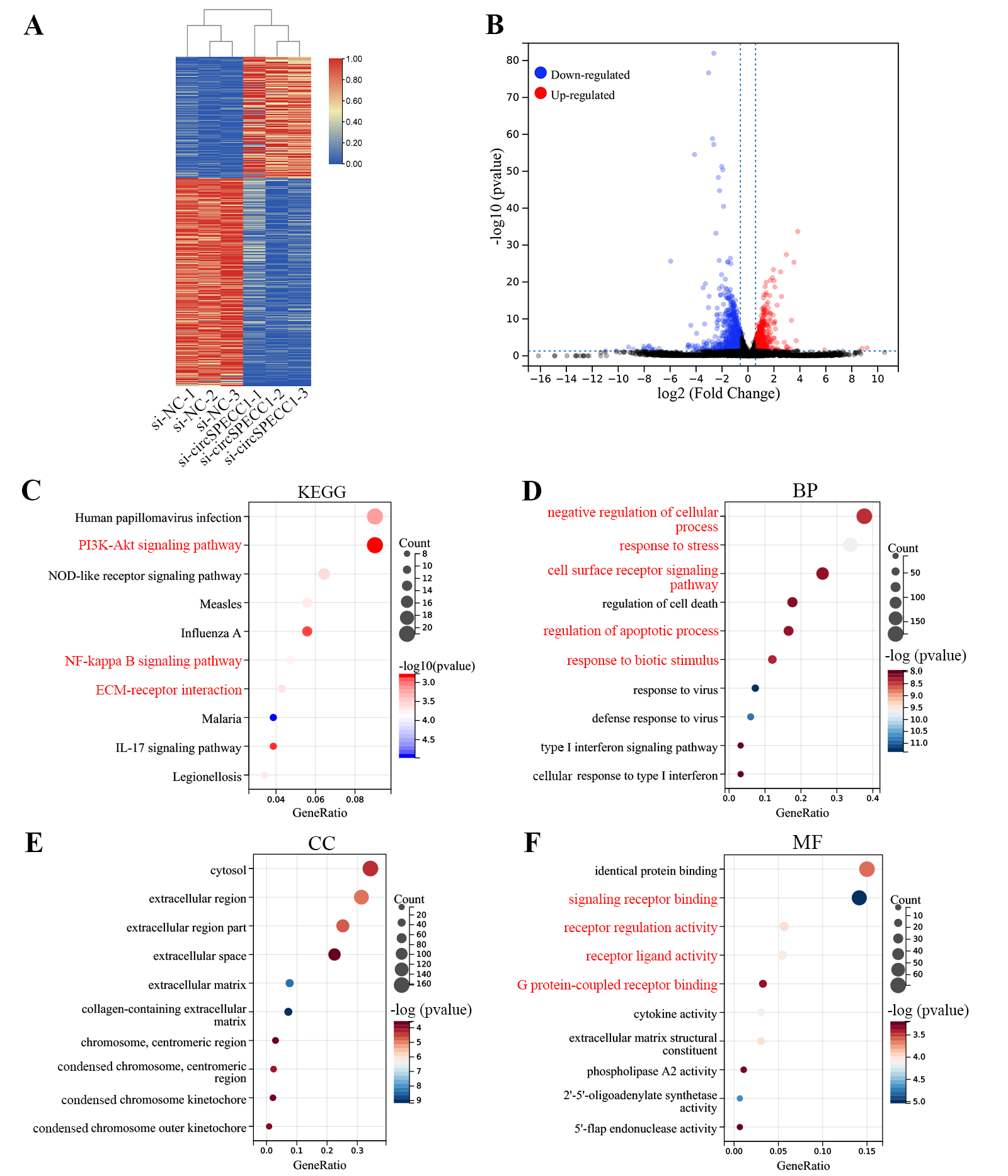


**Supplementary Figure 5. Transcriptome analysis after knockdown of circSPECC1 in LN229 cell line.**

(A) Following circSPECC1 knockdown, the cluster heatmap displayed the genes that were differentially expressed. (B) Following circSPECC1 knockdown, the volcano map displayed the differentially expressed genes (|FC|≤2, P<0.05, and FDR<0.05).

(C) KEGG enrichment analysis of genes that were differentially expressed in the LN229 cell line following circSPECC1 knockdown. GO enrichment analysis of differentially expressed genes after knocking down circSPECC1 in LN229 cell line, biological process (BP) (D), cellular component (CC) (E), and molecular function (MF) (F) enrichment analyses were included.


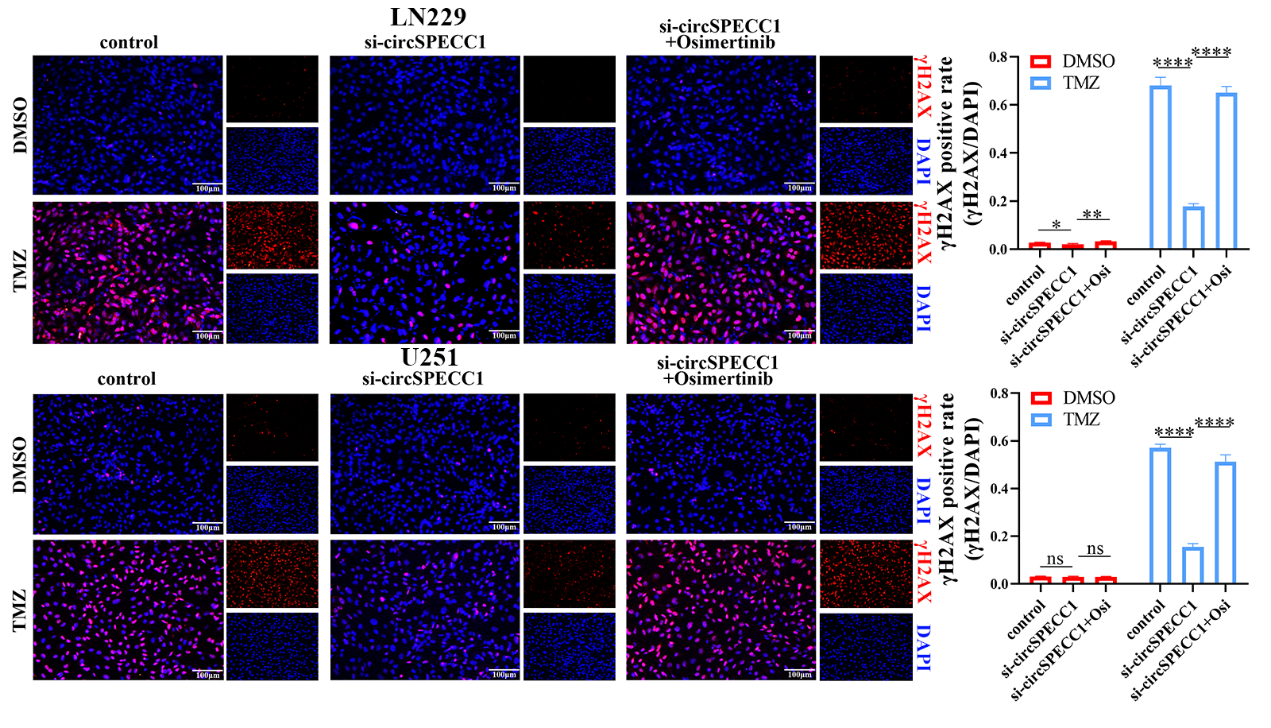


**Supplementary Figure 6. Osimertinib could restore the sensitivity of LN229 and U251 cells to TMZ after knocking down circSPECC1 via γH2AX immunofluorescence assay.** ns, *P*＞0.05; *, *P*＜0.05; **, *P*＜0.01. ****, *P*＜0.0001.
